# Evaluation of the effects of space allowance on measures of animal welfare in laboratory mice

**DOI:** 10.1038/s41598-017-18493-6

**Published:** 2018-01-15

**Authors:** Jeremy D. Bailoo, Eimear Murphy, Justin A. Varholick, Janja Novak, Rupert Palme, Hanno Würbel

**Affiliations:** 1 Division of Animal Welfare, University of Bern, Bern, CH Switzerland; 2 Department of Biomedical Sciences, University of Veterinary Medicine Vienna, Vienna, AT Austria

## Abstract

We studied how space allowance affects measures of animal welfare in mice by systematically varying group size and cage type across three levels each in both males and females of two strains of mice (C57BL/6ByJ and BALB/cByJ; n = 216 cages, a total of 1152 mice). This allowed us to disentangle the effects of total floor area, group size, stocking density, and individual space allocation on a broad range of measures of welfare, including growth (food and water intake, body mass); stress physiology (glucocorticoid metabolites in faecal boli); emotionality (open field behaviour); brain function (recurrent perseveration in a two-choice guessing task); and home-cage behaviour (activity, stereotypic behaviour). While increasing group size was associated with a decrease in food and water intake in general, and more specifically with increased attrition due to escalated aggression in male BALB mice, no other consistent effects of any aspect of space allowance were found with respect to the measures studied here. Our results indicate that within the range of conditions commonly found in laboratory mouse housing, space allowance as such has little impact on measures of welfare, except for group size which may be a risk factor for escalating aggression in males of some strains.

## Introduction

Although the mouse, *Mus musculus*, is the most widely used species of animal in laboratory research, surprisingly little is known about how the behavioural biology of the mouse relates to social and physical aspects of laboratory housing conditions^1^. Consequently, “space and housing needs” have been identified as major topics where empirical data are critically lacking^2–4^.

To regulate laboratory housing, mice are broadly categorized into breeding and non-breeding animals. This study focused on non-breeding mice and examples of legal guidelines regulating space allowance requirements for the housing of non-breeding mice are found in Supplementary Table 1a. Although it has been widely acknowledged that this may be inadequate, these guidelines are generally based on space allocation per mouse in relation to body mass^3^. However, evaluation of space needs remains difficult, as the legal guidelines contrast sharply with the available scientific evidence^2^.

Reviews of the existing literature have highlighted a relative dearth of empirical research as well as ambiguous results^2,5^. At least some of the discrepancies between studies have been attributed to the confounding effects of variation in either group size (i.e., number of mice per cage) or floor area (i.e., cage size). Stocking density (group size in a given floor area, e.g., 3 mice/300 cm^2^) and space allocation (floor area per mouse, e.g., 100 cm/mouse) are intimately related. If floor area is kept constant and group size is increased, stocking density is increased while space allocation is decreased. Conversely, if group size is kept constant and floor area is increased, stocking density is decreased while space allocation is increased.

Most of the previous studies investigating the space needs of laboratory mice have neglected this inverse relation and confounded space allocation and stocking density^2,6–10^. Moreover, the few studies investigating space allocation have focused on male mice with aggression, mortality, and attrition due to aggression as primary outcome variables. One study, which used space allocations larger than those typically found in laboratory settings (900 to 3800 cm^2^), found no differences in aggression^11^. Other studies which used space allocations more applicable to typical laboratory housing (30 to 129 cm^2^) have found either increased levels^10,12^ or no differences in aggression^6^, and strain specific effects^6,7^ as space allocation increased. Studies investigating stocking density are more numerous and generally indicate that as stocking density increases, aggressive behaviour increases^10,13^, although more variable results have been observed with respect to stress reactivity^2,14,15^.

Formal conclusions based on the extant literature are therefore limited, with current legal guidelines being informed by “common practice” rather than empirical evidence. A similar “common practice” policy is observed in Swiss legislation, where the space allowance for mice kept as pets is markedly higher than that for laboratory mice (Supplementary Table 1b).

Given the relative dearth of systematic empirical studies and the variable results reported by them, we refrained from formulating specific hypotheses, although by using inferential statistics we generally tested for evidence against the null hypotheses but without specification of the direction of the effects. Thus, the present study is exploratory in nature and systematically varies group size and floor area, and therefore stocking density and space allocation, in a full factorial design and assesses how variation in these parameters affects measures of welfare. Because animal welfare is not a unitary construct, we employed a multifaceted approach to its measurement, combining measures of home-cage behaviour, growth, stress physiology, and behavioural measures related to emotionality and brain function.

We used two strains of mice, C57BL/6ByJ and BALB/cByJ (hereafter, C57 and BALB), because they are two of the more common inbred strains of mouse in biomedical research and because they have been used in previous studies in this area^6,7,16^. These two reasons, together with the inclusion of both sexes, were means of increasing the generalizability and applicability of the results of this study.

## Method

### Design

**Figure 1:**
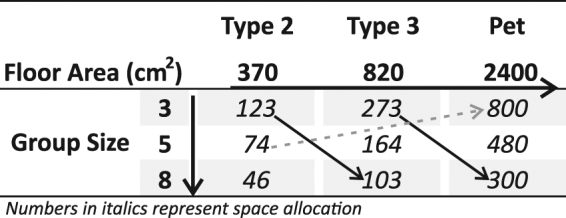
Schematic of experimental design, indicating group size, floor area, and space allocation. Numbers in italics represent space allocation.

This study employed a 2 (sex) × 2 (strain) × 3 (floor area) × 3 (group size) full factorial design. The experimental unit was the cage, and an *a priori* sample size calculation with an estimated medium effect size (Cohen’s *f* = 0.25) yielded 216 cages; 6 per factor constellation. Group size and floor area were varied across three levels to increase the generalizability of our conclusions concerning these parameters (Fig. 1). Floor area was varied using two of the more common laboratory cages for mice in Europe and Switzerland, Makrolon® Type 2 and Type 3 cages, and also using a “Pet” polycarbonate cage that mimicked the guidelines for housing pet mice in Switzerland (Supplementary Fig. 1).

**Figure 2:**
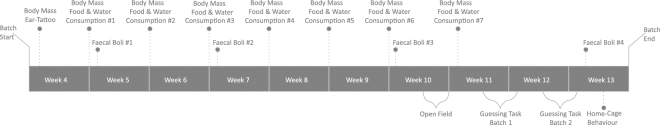
Experimental timeline for a single batch of animals.

**Figure 3:**
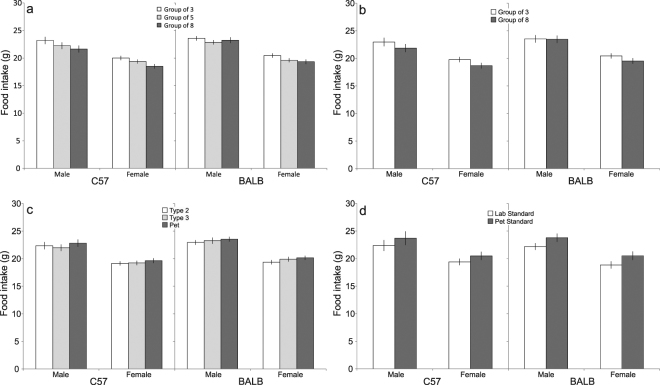
Variation in food intake (Estimated Marginal Means ± 95% CI) in relation to: (**a**) group size, while controlling for floor area; (**b**) group size, while keeping space allocation constant; (**c**) floor area, while controlling for group size; and (**d**) lab vs. pet standards.

This design permitted us to investigate whether measures of animal welfare are impacted by: (1) increasing group size while controlling for floor area (vertical arrow, Fig. 1); (2) increasing group size while controlling for space allocation (diagonal arrow, Fig. 1); (3) increasing floor area while controlling for group size (horizontal arrow, Fig. 1); and (4) differences between laboratory standards and pet standards (dashed arrow, Fig. 1).

We note that the comparison in which group size was increased and space allocation was held constant was not equal (123 vs 103 cm^2^ and 273 vs 300 cm^2^). However, as space allocation also varies depending on individual body mass and size across time, we believe that these differences do no compromise the validity of this comparison.

### Subjects

Six batches of 48 males and 48 females of newly weaned (21–25 days old at delivery) non-sibling C57 (n = 576) and BALB (n = 576) mice were ordered from Charles River, France. Four mice were not included in the experiment due to issues at delivery, and BALB males were excluded from the last two batches due to early termination criteria being reached by many cages in the first four batches (see section 2.5 and Supplementary Methods 1 for further details). All mice were ear-tattooed for identification upon arrival to the laboratory.

### Husbandry procedures

Animals were kept on a 12:12 h light:dark cycle with lights on at 22:00. A red light emitting diode (LED) light remained on throughout the entire cycle. Temperature was maintained at 22 ± 1 °C with an average humidity of 40%. All cages contained wood shavings 2 cm deep (Lignocel® select) and 10–11 grams of shredded paper for nesting material (Sizzle-Pet®). Animals had *ad libitum* access to standard rodent chow (Kliba Nafag #3430, Switzerland) and tap water. Cages and water bottles were changed once weekly in the dark phase under red light.

### Outcome variables

Data were collected across a range of measures, including (a) Growth (food and water intake, body mass); (b) Stress Physiology (glucocorticoid metabolites in faecal boli); (c) Emotionality (open field behaviour); (d) Brain Function (recurrent perseveration in a two-choice guessing task); and (e) Home-Cage Behaviour (activity, stereotypic behaviour) (Fig. 2). Only one randomly selected animal from each cage was tested in both the open field and the guessing task, and was later scored for home-cage behaviour. This focal animal was tail marked daily using a permanent marker (Edding 500), beginning at cage changes in week 9 and continuing until the end of the experiment.

#### Growth: Body mass and food and water consumption

Longitudinal assessment of food and water intake and body mass may be used as crude measures for assessing different types of stress, as acute stress is associated with a loss in body mass and chronic stress with a gain in body mass^17–20^. Food and water intake are necessarily coupled and positively correlated.

Beginning with the arrival of the animals at the laboratory and continuing until the beginning of food restriction for the guessing task (Fig. 2), the mass of the food and water remaining in each cage was subtracted from the mass of food and water provided to each cage at cage change. Thus, we measured food disappearance rather than food intake, as particulates of food in the bedding remained unaccounted for. For analysis, food and water consumption data were corrected for the number of animals per cage. The mass of each animal was also recorded at weekly cage changes.

#### Stress Physiology: Glucocorticoid metabolite analysis

Non-invasive methods of quantifying circulating levels of glucocorticoids, a primary product of the activation of the hypothalamus-pituitary-adrenal (HPA) stress system, are preferable to invasive methods such as blood sampling, because they do not elicit a stress response^21^. In mammals, glucocorticoids are metabolized by the liver and are excreted in both urine and faeces^22^. A method of analysing these metabolized by-products has been developed and validated^22^, and was therefore used as a measure of the stress experienced by the mice in our study.

Faecal boli were collected at four time points in the dark phase under red light approximately 24 hours after cage changes. A minimum of 2 boli per animal per cage was collected. Samples were immediately frozen at −20 °C and later processed blindly (JAV, JN, and RP) to assess the concentration of 5α-3β, 11β-corticosterone metabolites (ng/0.05 g faeces) according to the method described by Touma and colleagues^23,24^. Of the 775 samples collected, which excludes 17 samples due to early termination criteria being reached, 3 samples were not processed because insufficient sample material was available.

#### Emotionality: Open field behaviour

The open field apparatus has been used to identify and validate behavioural differences related to anxiety in both rats and mice^25,26^. More specifically, longitudinal assessment of the pattern of locomotor behaviour in the open field across repeated exposures has been demonstrated to provide information about how animals behaviourally cope with a stressor and how the HPA axis differentially operates between groups of animals^27,28^.

In the present experiment, four open field arenas were used to test mice in squads of four (see Supplementary Methods 2 for details). Total distance travelled and time spent in the centre of the arena were digitally recorded (960P, 30 fps) across four days between 10:00 to 13:00. Videos were processed within Noldus EthoVision XT such that each point was accurately scored and issues associated with automated tracking were eliminated^29^.

#### Brain Function: Recurrent perseveration (guessing task)

The expression of stereotypic behaviour has been found to be correlated with recurrent perseveration, a form of impaired inhibitory control of behaviour, both in humans (autistic children) and in captive mammals and birds. A positive correlation between stereotypy levels and recurrent perseveration has also been found in studies in mice, notably in C57 mice^30^, although other studies have yielded mixed results^31–34^.

To assess recurrent perseveration, we measured a perseveration score (logit [P]) and the frequencies of pure repetitions (LLLL, RRRR) and pure alternations (RLRL, LRLR) relative to all possible tetragrams of consecutive choices (n = 16) in a two-choice guessing task, using the same apparatus and a slightly modified procedure to Novak and colleagues^34^. These modifications were used to facilitate habituation to the apparatus and to ensure that mice were reliably eating the food rewards presented in the apparatus (see Supplementary Methods 3 for details).

#### Home-cage behaviour

To assess the expression of stereotypic behaviour and relate this to measures of recurrent perseveration, home-cage behaviour was recorded two days after completion of the guessing task. Stereotypies are the most prevalent form of abnormal repetitive behaviour in mice^35,36^, and conditions associated with other measures of poor welfare have previously been associated with the development of stereotypic behaviour in mice^1,37^.

Video recordings were scored using Noldus Observer XT (version 10.5) by JDB using a previously validated ethogram^38^ (Supplementary Table 3). Videos were first screened for general activity and, based on these data, mice were observed for 15 minutes during the 2^nd^ and 4^th^ hour of the dark phase. Using one-zero sampling with 15 s intervals, yielding 120 data points per mouse, the videos were sampled for the occurrence of the different types of stereotypy and the proportion of intervals of active time that each stereotypy was observed. One video per strain per sex per batch was pseudo-randomly selected and re-scored to assess intra-rater reliability.

### Attrition

Our laboratory employs a scoring system for daily assessment of rodent health along with specific termination criteria based on humane endpoints (Supplementary Fig. 5). Unlike previous studies^6,7,16^, the onset of aggression in BALB male mice was reliably observed at 8 weeks of age in each batch. This was accompanied by increasing levels of cloudiness of the walls of the cages, possibly due to increased contact during aggressive encounters (*personal observation, JDB, EM*). Of the 36 cages of BALB males used in this experiment, 20 cages (56%) reached early termination criteria by 10 weeks of age. The progression to termination involved unkemptness of fur (lack of grooming), hunched posture of animals, decreased mobility, and wounding.

One female C57 mouse in batch 4, aged 8 weeks, was euthanized because of malocclusion and two BALB males were found dead (batch 2, aged 4 weeks and batch 4, aged 10 weeks, respectively).

### Ethical Statement

This study was carried out in accordance with the guidelines of the Swiss Animal Welfare Ordinance (TSchV 455.1). It was approved by the Cantonal Veterinary Office in Bern, Switzerland (permit number: BE4/14).

### Statistical Analysis

With the exception of home-cage and stereotypic behaviour, all statistical analyses were performed using the MIXED procedure with IBM^TM^ SPSS Statistics (version 23). Assumptions of normally distributed errors and homogeneity of variance were examined graphically and, based on these inspections; no transformations of data were needed. Home cage and stereotypic behaviour was analysed using Kruskall-Wallis independent sample tests or Mann-Whitney U tests, due to the high degree of skewness observed in the data. Strains of mice were analysed separately because of the high degree of observed attrition for BALB males.

For outcome measures with repeated measurement, the predictor “age” was included as a covariate. To evaluate the effects of increasing group size while controlling for floor area, a full model with group size and sex as fixed predictors and cage type as a random effect was run. To evaluate the effect of increased group size while controlling for space allocation, animals housed in groups of 3 in Type 2 and Type 3 cages were compared to animals housed in groups of 8 in Type 3 and Pet cages — a full model including sex as a fixed factor was run. To evaluate the effects of increasing floor area while controlling for group size, a full model with cage type and sex as fixed factors and group size as a random effect was run. Finally, differences between laboratory standards and Pet standards were evaluated by comparing animals housed in groups of 5 in Type 2 cages with animals housed in groups of 3 in Pet cages — a full model including sex as a fixed factor was run. P-values less than 0.05 were considered statistically significant for all analyses. Bonferroni corrections were applied to all subset analyses within outcome measure as well as to all *post hoc* comparisons of significant main effects and interactions. Raw data for all outcome measures will be made available upon request.

## Results

### Growth: Food intake per mouse

Controlling for floor area, group size significantly affected food intake in both C57 and BALB mice (C57: *F*_2,99_ = 18.68, *p*  < 0.05; BALB: *F*_2,84_ = 3.80, *p* <0.05), indicating that food intake decreased with increasing group size. This was confirmed by *post hoc* comparisons revealing that groups of 3 ate more than larger groups in C57 mice, while in BALB mice, groups of 3 ate more than groups of 5 (Fig. 3a). Holding space per mouse constant, a similar effect of group size was found in C57 mice only, where mice housed in groups of 3 ate more than mice housed in groups of 8 (C57: *F*_1,57_ = 19.89, *p* <0.05; BALB: *F*_1,37_ = 1.55, *p* > 0.05; Fig. 3b). When controlling for group size, increasing floor area had no effect on food intake in either strain (C57: *F*_2,99_ = 2.69, *p* > 0.05; BALB: *F*_2,83_ = 2.56, *p* > 0.05; Fig. 3c). However, in both strains, mice housed at pet standards ate more than mice housed at lab standards (C57: *F*_1,36_ = 16.45, *p*  < 0.05; BALB: *F*_1,25_ = 21.35, *p* <0.05; Fig. 3d).

Effects of sex, age, and an interaction between sex and age were found in both strains which indicated that the difference in food intake between male and female mice varied across weeks of age (Supplementary Table 7, Supplementary Fig. 6).

### Growth: Water intake per mouse

**Figure 4:**
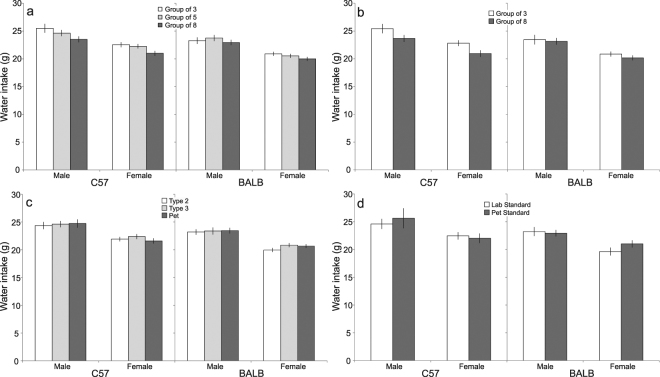
Variation in water intake (Estimated Marginal Means ± 95% CI) in relation to: (**a**) group size, while controlling for floor area; (**b**) group size, while controlling for space allocation; (**b**) floor area, controlling for group size; and (**d**) lab vs. pet standards.

Similar to food intake, water intake decreased with increasing group size, although this effect was significant in C57 mice only. Thus, when controlling for floor area, group size significantly affected water intake in C57 mice (C57: *F*_2,100_ = 17.64, *p* < 0.05; Fig. 4a). *Post hoc* comparisons revealed that mice housed in groups of 3 drank more than larger groups. By contrast, no effect of group size was found in BALB mice (BALB: *F*_2,84_ = 1.03, *p* > 0.05; Fig. 4a). By holding space per mouse constant, a similar effect of group size was found in C57 mice, where mice housed in groups of 3 drank more than mice housed in groups of 8; a difference that was not observed in BALB mice (C57: *F*_1,45_ = 19.94, *p*  < 0.05; BALB: *F*_1,37_ = 1.55, *p* >  0.05; Fig. 4b). In both strains, decreasing floor area while controlling for group size had no effect on water intake (C57: *F*_2,99_ = 0.32, *p* > 0.05; BALB: *F*_2,83_ = 1.53, *p* > 0.05; Fig. 4c). Furthermore, water intake did not differ between mice housed using pet or lab standards, respectively (C57: *F*_1,39_ = 0.23, *p* > 0.05; BALB: *F*_1,18_ = 0.79, *p* > 0.05; Fig. 4d).

Effects of sex and age were found in both strains, and an interaction between sex and age was additionally observed in BALB mice (Supplementary Table 7). For C57 mice, male mice drank more than female mice, and water intake increased with age. In BALB mice, the difference in water intake between male and female mice additionally varied across weeks of age (Supplementary Fig. 7).

### Growth: Body mass

**Figure 5:**
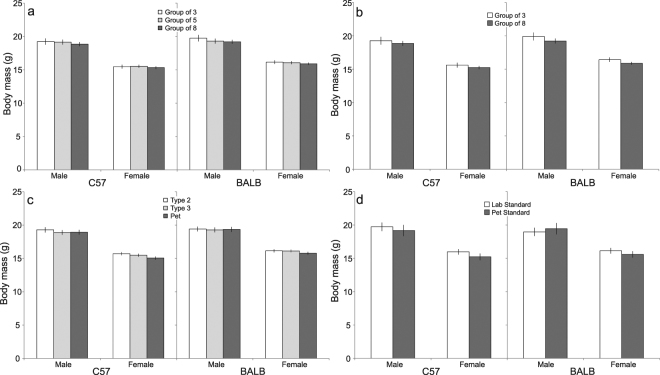
Variation in body Mass (Estimated Marginal Means ± 95% CI) in relation to: (**a**) group size, while controlling for floor area; (**b**) group size, while keeping space allocation constant; (**c**) floor area, while controlling for group size; and (**d**) lab vs. pet standards.

Despite the effects on food and water intake, there was no main effect of group size on body mass in either strain when controlling for floor area (C57: *F*_2,635_ = 2.05, *p* > 0.05; BALB: *F*_2,513_ = 1.31, *p* > 0.05; Fig. 5a). However, in C57 mice, a significant interaction between age and group size was observed; mice housed in groups of 8 weighed less than groups of 5 at arrival, and less than groups of 3 and 5 in the following week but did not differ thereafter (*F*_7,3566_ = 133.17, *p* < 0.05; Supplementary Figure 8). Holding space per mouse constant, increasing group size had no effect on body mass in either strain (C57: *F*_1,48_ = 1.74, *p* > 0.05; BALB: *F*_1,45_ = 3.35, *p* > 0.05; Fig. 5b). In contrast, when controlling for group size, increasing floor area resulted in a decrease in body mass, but this effect was significant in C57 mice only (C57: *F*_2,637_ = 5.41, *p*  < 0.05; BALB: *F*_2,516_ = 1.00, *p* > 0.05; Fig. 5c). Additionally, a significant interaction between floor area and age reflects that from week 7, C57 mice housed in the smallest cages weighed more than those in larger cages (C57: *F*_14,3567_ = 2.00, *p* < 0.05; Supplementary Fig. 9a). While a similar interaction was found for BALB mice (BALB: *F*_14,3026_ = 1.78, *p* < 0.05), *post hoc* comparisons yielded no significant differences after correction for multiple testing (Supplementary Fig. 9b). Body mass did not differ between mice housed according to pet or lab standards, respectively (C57: *F*_1,26_ = 3.83, *p* > 0.05; BALB: *F*_1,15_ = 0.00, *p* > 0.05; Fig. 5d).

Effects of sex, age, and an interaction between sex and age were found in both strains which indicated that male mice weighed more than female, that body mass increased with age, and that the difference between male and female mice varied across age (Supplementary Table 7, Supplementary Fig. 10).

### Stress Physiology: Faecal glucocorticoid metabolite concentration

There was no effect of increasing group size on glucocorticoid metabolite concentration for either strain when controlling for floor area (C57: *F*_2,104_ = 0.35, *p* > 0.05; BALB: *F*_2,122_ = 0.67, *p* > 0.05; Supplementary Fig. 11a), nor when holding space per mouse constant (C57: *F*_1,40_ = 1.36, *p* > 0.05; BALB: *F*_1,50_ = 0.87, *p* > 0.05; Supplementary Fig. 11b). Similarly, glucocorticoid metabolite concentration in both strains was not affected by either increasing floor area when holding group size constant (C57: *F*_2,105_ = 0.51, *p* > 0.05; BALB: *F*_2,119_ = 0.48, *p* > 0.05; Supplementary Fig. 11c), or when comparing mice housed according to pet and lab standards, respectively (C57: *F*_1,25_ = 0.03, *p* > 0.05; BALB: *F*_1,17_ = 0.12, *p* > 0.05; Supplementary Fig. 11d).

Glucocorticoid metabolite concentration varied by sex, age, and as an interaction between sex and age in both strains (Supplementary Table 7). Female C57 mice had lower levels of glucocorticoid metabolites in week 5, but higher levels in weeks 7 and 10 compared to males, while BALB females had significantly higher levels of glucocorticoid metabolites across all measured time points compared to males (Supplementary Fig. 12).

### Emotionality: Open Field Behaviour

Increasing group size had no effect on the behaviour of mice in the open field when controlling for floor area, (*distance travelled* C57: *F*_2,112_ = 0.67, *p* > 0.05; BALB: *F*_2,91_ = 0.41, *p* > 0.05; Supplementary Fig. 13a; *time-in-centre* C57: *F*_2,112_ = 0.54, *p* > 0.05; BALB: *F*_2,91_ = 0.24, *p* > 0.05; Supplementary Fig. 14a), or when holding space per mouse constant (*distance travelled* C57: *F*_1,47_ = 3.64, *p* > 0.05; BALB: *F*_1,39_ = 2.96, *p* > 0.05; Supplementary Fig. 13b; *time-in-centre* C57: *F*_1,51_ = 0.89, *p* > 0.05; BALB: *F*_1,39_ = 1.13, *p* > 0.05; Supplementary Fig. 14b). Similarly, increasing floor area while holding group size constant also had no effect on open field behaviour in either strain (*distance travelled* C57: *F*_2,110_ = 0.73, *p* > 0.05; BALB: *F*_2,90_ = 1.07, *p* > 0.05; Supplementary Fig. 13c; *time-in-centre* C57: *F*_2,113_ = 0.82, *p* > 0.05; BALB: *F*_2,90_ = 0.51, *p* > 0.05; Supplementary Fig. 14c). No differences were observed between mice housed in lab and pet cages (*distance travelled* C57: *F*_1,47_ = 3.64, *p* > 0.05; BALB: *F*_1,39_ = 2.96, *p* > 0.05; Supplementary Fig. 13d; *time-in-centre* C57: *F*_1,51_ = 0.89, *p* > 0.05; BALB: *F*_1,39_ = 1.13, *p* > 0.05; Supplementary Fig. 14d).

C57 mice showed habituation in behaviour in the open field across days of testing, whereas sex and the interaction between day and sex were not significant (Supplementary Table 7). Thus, both in male and female C57 mice the distance travelled and the time spent in the centre of the open field decreased across days of testing (Supplementary Fig. 15). In contrast, BALB mice showed sensitization in distance travelled across days of testing, and habituation in time spent in the centre of the open field. Thus, distance travelled increased across days, both in males and females, whereas time spent in the centre of the open field decreased across days of testing. Furthermore, in BALB mice behaviour in the open field varied as a consequence of sex and an interaction between sex and time, indicating that males travelled over longer distances but spent less time in the centre of the open field, and that these differences between males and females varied across days of testing (Supplementary Table 7, Supplementary Fig. 16).

### Brain Function: Guessing Task

No effect of group size was found on the responses of mice in the guessing task when controlling for floor area (*perseveration score* C57: *F*_2,98_ = 2.48, *p* > 0.05; BALB: *F*_2,69_ = 0.22, *p* > 0.05; Supplementary Fig. 17a; *repetitions and alternations* C57: *F*_2,1656_ = 0.39, *p* > 0.05; BALB: *F*_2,1192_ = 2.34, *p* > 0.05; Supplementary Fig. 18a). Increasing group size while holding space per mouse constant, however, did have an effect on perseverative behaviour in C57 mice, with mice housed in groups of 8 showing higher perseveration scores than those housed in groups of 3. No such effect was found in BALBs (*perseveration score* C57 *F*_1,42_ = 5.12, *p* <0.05; BALB: *F*_1,28_ = 0.09, *p* > 0.05; Supplementary Figure 17b). The distribution of choices were unaffected by increasing group size (*repetitions and alternations* C57: *F*_1,730_ = 1.27, *p* > 0.05; BALB: *F*_1,506_ = 0.20, *p* > 0.05; Supplementary Fig. 18b). The responses of mice in the guessing task were neither affected by increasing floor area while holding group size constant (*perseveration score* C57: *F*_2,96_ = 0.23, *p* > 0.05; BALB: *F*_2,69_ = 0.01, *p* > 0.05; Supplementary Fig. 17c; *repetitions and alternations* C57: *F*_2,1656_ = 0.06, *p* > 0.05; BALB: *F*_2,1192_ = 1.78, *p* > 0.05; Supplementary Fig. 18c), nor when comparing mice housed in lab versus pet standards (*perseveration score* C57: *F*_1,20_ = 0.25, *p* > 0.05; BALB: *F*_1,15_ = 0.09, *p* > 0.05; Supplementary Fig. 17d; *repetitions and alternations* C57: *F*_1,378_ = 0.19, *p* > 0.05; BALB: *F*_1,298_ = 0.02, *p* > 0.05; Supplementary Fig. 18d).

Perseverative behaviour was not related to sex in either strain (Supplementary Table 7). In both strains, the distribution of choices was not equal — C57 mice performed more pure alternations (LRLR and RLRL) while BALB mice performed more pure repetitions (LLLL, RRRR) relative to the other possible sequences (Supplementary Table 7, Supplementary Fig. 19).

### Home-cage behaviour of focal animals

Of the 198 cages of animals used in this experiment, 178 cages were available for behavioural screening at the end of the experiment (91%). Of the missing cages, 19 were male BALB cages, and 1 was a male C57 cage. Intra-rater reliability was calculated in Noldus Observer and was found to be high, κ = 0.87^39^.

Activity levels in the home-cage did not differ between treatments (*group size*: *H* = 3.40, *p* > 0.05; *cage type*: *H* = 2.73, *p* > 0.05). C57 mice were active in 95 ± 11% of the observed intervals, while BALB mice were active in 93 ± 14% of the observed intervals. Activity in the observed intervals did not vary by sex for either strain (C57: *U*  =  1.42, *p* > 0.05; BALB: *U* = 0.41, *p* > 0.05).

The proportion of intervals in which mice were active did not differ with increasing group size, without controlling for floor area, in either strain or sex (C57 male: *H* = 0.70, *p* > 0.05; C57 female: *H* = 1.86, *p* > 0.05; BALB male: *H = *10.65, *p* > 0.05; BALB female: *H = *0.08, *p* > 0.05), or when holding space per mouse constant (C57 male: *U* = −0.39, *p* > 0.05; C57 female mice: *U* =−1.00, *p* > 0.05; BALB male: *U* = −1.01, *p* > 0.05; BALB female: *U* = 0.94, *p* > 0.05). Similarly, no differences in activity levels were found with increasing floor area, without controlling for group size, (C57 male: *H* = 0.89, *p* > 0.05; C57 female: *H* = 2.90, *p* > 0.05; BALB male: *H* = 4.21, *p* > 0.05; BALB female: *H* = 2.58, *p* > 0.05), or when comparing mice housed in lab and pet standards (C57 male: *U* = 0.36, *p* > 0.05; C57 female: *U* = 1.00, *p* > 0.05; BALB male: *U* = 0.00, *p* > 0.05; BALB female: *U*= 0.86, *p* > 0.05).

Of the 178 focal mice screened for stereotypic behaviour, 112 mice exhibited no stereotypic behaviour (63%, Supplementary Table 4). More BALB mice performed stereotypic behaviour than C57 mice (76% vs. 11%). BALB mice performed mostly bar-mouthing stereotypies (89%) while route-tracing was the most prevalent stereotypy in C57 mice (83%).

The level of stereotypic behaviour (number of intervals where stereotypic behaviour was observed) did not differ with increasing group size, without controlling for floor area, in either strain or sex (C57 male: H = 3.64, p > 0.05; C57 female: H = 4.73, *p* > 0.05; BALB male: H = 2.91, p > 0.05; BALB female: H = 1.48, p > 0.05), or when holding space per mouse constant (C57 male: U = 1.81, p > 0.05; C57 female mice: U = 0.20, p > 0.05; BALB male: U = −1.5, p > 0.05; BALB female: U = 1.39, p > 0.05). Similarly, no differences in activity levels were found with increasing floor area, without controlling for group size, (C57 male: H = 3.67, *p* > 0.05; C57 female: H = 1.65, *p* > 0.05; BALB male: H = 3.83, *p* > 0.05; BALB female: H = 1.61, *p* > 0.05), or when comparing mice housed in lab and pet standards (C57 male: U = 1.06, *p* > 0.05; C57 female: U = 1.48, *p* > 0.05; BALB male: U = 0.29, *p* > 0.05; BALB female: U = 0.33, *p* > 0.05).

On average, the level of stereotypic behaviour was higher in females for the more pronounced types of stereotypy (BALB, bar-mouthing; C57, route-tracing; Supplementary Table 5). The average level of stereotypic behaviour for both male and female BALB mice was similar (19%) compared to C57 mice that displayed disproportionate average levels of total stereotypic behaviour between sexes (Males = 2%, Females = 15%).

### Attrition due to aggression

The levels of attrition due to aggressive behaviour in male BALB mice was unexpected based on previous studies^6,7,16^. Based on overall attrition, the observed distribution of attrition across treatment groups in BALB males was significantly different from what would be expected by chance alone (*χ*^2^(4) = 34.29, Supplementary Table 6). The distribution of attrition varied with increasing group size (*χ*^2^(2) = 32.14) and with increasing floor area (*χ*^2^(2) = 11.78) with the lowest level of attrition being observed in the smallest group size housed in the smallest floor area.

## Discussion

To study the effects of space allowance in all of its facets on measures of animal welfare in mice, we systematically varied group size and cage type across three levels each, using a full factorial design. This allowed us to disentangle effects of total floor area, group size, stocking density and individual space allocation on measures of welfare. Our results show that space allowance, as such, had little effect on growth, HPA stress response, emotionality, inhibitory control of behaviour (perseveration), and abnormal repetitive behaviour (stereotypies) in the home-cage. The only consistent effects were a decrease in food and water intake with increasing group size and increased attrition caused by escalated aggression with increasing group size and floor area in male BALB mice (c.f., Table 1).

**Table 1:** Summary of observed differences between treatments and outcomes used in this study.

Outcome Measure	*Increasing group size (3,5,8), controlling for floor area*		*Increasing group size (3,8), controlling for space allocation*		*Increasing floor area (370, 820, 2400* *cm*^2^), *controlling for group size*		*Comparing lab and pet standards in Switzerland*	
	C57	BALB	C57	BALB	C57	BALB	C57	BALB
Food intake	*Groups of 3 ate more*	*Groups of 3 ate more than groups of 5*	*Groups of 3 ate more*	*n.d*.	*n.d*.	*n.d*.	*Mice housed under Pet standards ate more*	*Mice housed under Pet standards ate more*
Water intake	*Groups of 3 and 5 drank more than groups of 8*	*n.d*.	*Groups of 3 drank more*	*n.d*.	*n.d*.	*n.d*.	*n.d*.	*n.d*.
Body weight	*Groups of 8 weighed less in the first two weeks*	*n.d*.	*n.d*.	*n.d*.	*Animals housed with 370* *cm*^2^ *weighed more from weeks 7–10*	*n.d*.	*n.d*.	*n.d*.
Glucocorticoid metabolite concentrations	*n.d*.	*n.d*.	*n.d*.	*n.d*.	*n.d*.	*n.d*.	*n.d*.	*n.d*.
Open Field: distance travelled	*n.d*.	*n.d*.	*n.d*.	*n.d*.	*n.d*.	*n.d*.	*n.d*.	*n.d*.
Open Field: time in centre	*n.d*.	*n.d*.	*n.d*.	*n.d*.	*n.d*.	*n.d*.	*n.d*.	*n.d*.
Guessing Task: perseveration score	*n.d*.	*n.d*.	*Groups of 8 more perseverative*	*n.d*.	*n.d*.	*n.d*.	*n.d*.	*n.d*.
Guessing Task: distribution of tetragrams	*n.d*.	*n.d*.	*n.d*.	*n.d*.	*n.d*.	*n.d*.	*n.d*.	*n.d*.
Stereotypic behaviour	*n.d*.	*n.d*.	*n.d*.	*n.d*.	*n.d*.	*n.d*.	*n.d*.	*n.d*.

*n.d* = no observed differences.

Food intake decreased with increasing group size when controlling for both floor area (cage type) and individual space allocation, although the effect was more variable in BALB mice. The negative relationship between group size and food intake is further supported by the higher food intake in mice housed under pet standards compared to those housed under lab standards. In this comparison, housing standard was confounded with group size; the pet standard was based on groups of 3 mice housed in pet cages while the lab standard was based on groups of 5 mice housed in type 2 cages. This difference in food intake between mice housed under pet and lab standards, respectively, was most likely a consequence of group size rather than floor area, as floor area had no effect on food intake when we controlled for group size. A similar, albeit weaker and less consistent relationship was found between group size and water intake. This relationship was not surprising given that both food and water intake are tightly linked to metabolism^40,41^ — although others have failed to find consistent effects on food and water intake^6,7^. Taken together, the effect of group size on food and water intake most likely reflects variation in metabolic demand. That group size rather than floor area had an effect on food (and water intake) further suggests that the effect was mediated by variation in microclimate (ambient temperature) rather than physical activity^13^. Importantly, as ambient temperatures in mouse facilities are kept below the thermoneutral zone of mice, resulting in cold stress^40,42^, increasing housing density may act as buffer. For example, mice housed in groups of 5 showed a preference for cooler temperatures (~1 °C lower) when inactive, compared to mice housed singly^42^. Therefore, the metabolic demand for maintaining homeostasis may be higher in smaller groups of mice. However, we were unable to examine this hypothesis further, as we did not measure temperatures inside the cages, and home-cage observations were deliberately timed to the most active time periods, when there was little variation in activity between strains, sexes, and treatment groups.

Despite the effect of group size on food intake, we did not find concordant increases in body mass with increasing group size. Moreover, in C57 mice, body mass decreased with increasing floor area (while controlling for group size), suggesting that body mass may depend more on physical activity, which generally increases with increasing cage size^2,14^. However, as mentioned above, our data did not allow us to examine this hypothesis further.

Neither measures of emotionality in the open field test nor glucocorticoid metabolite concentrations as a measure of the activation of the HPA stress system by the animals’ housing conditions were affected systematically by our treatments. To our knowledge, only a few studies have assessed effects of group size or floor space on behaviour in the open field^9,13,43^, and only one other study has assessed faecal glucocorticoid metabolite concentrations in relation to space allowance^13^. However, treatment differences in terms of group size and space allocation, as well as methodological differences in open field assessment, make the comparison of results between studies difficult. For example, one similar study^9^ comparing group sizes of 2,5, and 10 in C57 and BALB mice found that mice housed at lower densities were less exploratory than those housed at higher densities – although this difference was not consistent across time and by strain. Methodologically, that study^9^ confounded space allocation and stocking density, and in addition, compared behaviour in the open field across more widely spaced intervals than the present study.

There was also no effect of space allowance on the expression of stereotypic behaviour. Stereotypies in mice are associated with barren housing conditions which, by definition, lack relevant resources for the expression of highly motivated behaviour^35^. Simple forms of enrichment, such as adequate nesting material, may attenuate the prevalence of observed stereotypic behaviour^33,36^. We observed relatively low levels of stereotypic behaviour, particularly in C57 mice and the level of stereotypic behaviour was unrelated to our treatment conditions. This may simply have been a consequence of the fact that all of our cages were provided with nesting material^33^. In line with this, we did not find consistent effects of our treatments on recurrent perseveration in the two-choice guessing task. There was an effect of group size on perseveration in C57 mice when holding space per mouse constant; thus, C57 mice housed in groups of 8 were more perseverative than those housed in groups of 3. However, given the large variation in the data and the relatively low perseveration scores compared to other studies in mice^32^, as well as the absence of higher levels of patterned responding (tetragrams of pure repetitions and alternations), this result may be a statistical artefact rather than biologically meaningful.

The other consistent treatment effect besides the effect of group size on food (and water) intake was an increase in escalating aggression, leading to attrition with increasing group size in male BALB mice. Although male BALB mice are known to be aggressive^44^, no other study using this strain has reported as high levels of attrition as observed in this study^7,9,10,13^. However, in those studies, endpoints and attrition criteria other than death were not specified. Furthermore, van Loo and colleagues^10^ also reported that aggression in laboratory mice was affected by group size but not by cage size.

There were several effects of sex, but no sex by treatment interactions, on the measured outcomes. As expected, food and water intake were higher in males than females in both strains, and correspondingly males weighed more than females. When comparing glucocorticoid metabolite concentrations, female mice on average had higher levels of glucocorticoid metabolites than male mice. This difference was large in BALB mice across all measured time points, whereas in C57 mice, it was smaller and less consistent across the four measured time points. This sex difference is consistent with previous studies reporting higher levels of glucocorticoid metabolites in female mice^13,23,24^ and is not a biologically relevant difference but rather a methodological artefact. Specifically, the enzyme immunoassay used to quantify glucocorticoid metabolites in faeces exhibits higher cross reactivity with metabolites secreted from females than males, and so a direct comparison between sexes is impossible^23,24^. When comparing females across strains, BALB females displayed approximately 3 times higher glucocorticoid metabolite concentrations compared to C57 females suggesting that regardless of treatment condition, female BALB mice are “more stressed” by laboratory housing conditions that female C57 mice. Similar differences between these two strains, albeit of varying magnitudes, have previously been reported in the literature^13,45^. It is unlikely that these data are artefactual, as this relative difference was observed across all time points and because the mean interassay co-efficient of variation for these samples was low (i.e., ±3.64%).

In the open field test, we observed a striking strain difference: C57 mice displayed a general habituation response, with distance travelled decreasing across days and a corresponding decrease in the time spent in the centre of the open field, a phenotype which is associated with lower levels of anxiety and stress^26,28,46^. In contrast, BALB mice displayed increased levels of exploration and decreased amounts of time spent in the centre of the field across the four days of testing, a phenotype that is associated with higher levels of anxiety/stress^26,46^.

There was also a large difference between the two strains in the expression of stereotypic behaviour, with more BALB mice exhibiting stereotypic behaviour than C57 mice, and consequently higher overall levels of stereotypic behaviour in BALB mice compared to C57 mice. Whereas there was no apparent difference between male and female BALB mice, in C57 mice more females expressed stereotypic behaviour, resulting in higher levels of stereotypy in female C57 compared to male C57 mice. Overall, however, levels of stereotypic behaviour were relatively low in this study, which may be a consequence of the nesting material provided to all cages, as discussed above.

There was also a strain difference in the response sequences of C57 and BALB mice in the two-choice guessing task. In both strains, the distribution of the 16 different tetragrams of response sequences differed from a random distribution. Whereas C57 mice showed higher levels of pure alternations (LRLR, RLRL), BALB mice performed more pure repetitions (LLLL, RRRR). This strain difference in response strategy was consistent across both sexes, indicating that it may reflect an underlying strain difference in terms of patterned responding.

Overall, these observed strain differences between C57 and BALB mice across our different measures are consistent with previous reports. In particular, relative to C57 mice, BALB mice are more stress reactive, display higher levels of aggression and decrements in cognitive performance^13,45,47–49^.

In summary, these data indicate that variation in group size, floor area, stocking density or individual space allocation within the range of space allowance commonly used in mouse research may not strongly affect the behaviour and welfare of laboratory mice, at least in regards to the outcomes measured in this study, with the exception of increasing group size (and possibly cage size) which may be a risk factor for escalating aggression in males of some strains of mouse. These findings highlight the difficulty of making general recommendations for mice, given the observed strain and sex differences. These results are in line with reviews of studies investigating these parameters^2,5,50^. However, similar to previous reviews, we must echo caution in the interpretation of these findings. First, the present study, as well as previous studies, have focused on relatively crude measures of animal welfare. Further studies may be needed using methods to assess the animals’ emotional states (e.g., cognitive bias^51^) depending on specific housing standards. Second, albeit covering most of the range typically found in mouse studies, the range of space allowance, and in particular group size, studied here covers a relatively narrow range of conditions. Thus, the lack of more pronounced effects may indicate that variation within this narrow range may not matter much to mice. This, however, does not necessarily mean that current space regulations are appropriate, as our results only permit conclusions about relative levels of welfare between the studied treatments, but not about absolute levels of welfare.

Nevertheless, there are good reasons to assume that space allowance *per se* may not be the most important aspect of housing conditions for the welfare of laboratory mice. First, similar studies investigating effects of space allowance in mice^6–10,12–14,16^, as well as other rodents^52^ have found similar results. Second, other studies varying both space allowance and environmental enrichment generally found that the provision of critical resources (e.g., shelter, nesting material) had stronger effects on measures of welfare than the provision of extra space^33,53,54^. Therefore, further studies are needed which investigate the effects of floor area and group size in combination with other relevant variables, such as structural enrichments that promote active engagement with, and control over, the environment. This may be key in mediating social behaviour in ways that prevent escalating aggression, leading to high levels of stress, injury, and ultimately attrition^10,11,44^. Before we accept last resorts such as housing males at higher densities^10^ or singly to attenuate or prevent escalating aggression, we should first examine more systematically whether it is possible to create housing conditions for mice that respect their behavioural biology without compromising their welfare or the validity of the research conducted with them.

## Electronic supplementary material

Supplementary Information
