# Supplementary material for: Evaluation of the effects of space allowance on measures of animal welfare in laboratory mice
Source: Sci Rep. 2018 Jan 15;8:713. doi: 10.1038/s41598-017-18493-6 (PMC5768730; doi:10.1038/s41598-017-18493-6)
Supplement: Supplementary file 1 — Supplementary Information [file 41598_2017_18493_MOESM1_ESM.pdf]

Evaluation of the effects of space allowance on measures of animal welfare in laboratory mice.

Jeremy D. Bailoo[1], Eimear Murphy[1], Justin A. Varholick[1],

Janja Novak[1], Rupert Palme[2] and Hanno Würbel[1]

[1]*Division of Animal Welfare, University of Bern, CH*

[2]*Department of Biomedical Sciences, University of Veterinary Medicine Vienna, AT*

Supplementary Information:
Number of pages: 27
Number of figures: 19
Number of tables: 8

Supplementary Methods 1: Subjects

a) Ordering and delivery

Six batches of 48 males and 48 females of newly weaned (21-25 days old at delivery) non-sibling C57 and BALB mice were ordered from Charles River, France (n = 1152). The batches were delivered at 5 week intervals except for batches 3 and 4 which were spaced 14 weeks apart due to the winter break and construction work (c.f., SI Table 2).

b) Missing subjects at delivery

One male C57 subject died during individual marking, possibly due to a combination of weaning, transport, and restraint stress. The following shipping errors were made by the supplier: 1) batch 2 contained 1 mis-sexed BALB female, 2) one C57 female was not delivered in batch 3, and 3) batch 5 contained 1 mis-sexed BALB female and 1 BALB female with an injured leg. The latter animal was treated with Metacam® subcutaneously once daily for three days and recovered. She was included in the experiment but was not tested on our behavioural outcome measures. Additionally, due to persistent aggression among BALB males, with many males reaching early termination criteria, BALB males were excluded from the last two replicates. Thus, the total number of animals delivered was 1052 mice (SI Table 2).

c) Sorting and assignment of subjects

The experiment was conducted in two housing rooms separated by a testing room (Supplementary Figure 2). Each batch contained one Type 2, Type 3, and Pet cage, housing 3, 5 or 8 animals of each sex and strain, respectively. Each batch of mice was delivered in two boxes (per strain, per sex) containing 24 animals. Animals in each batch were allocated to each treatment group sequentially so that individual differences as a consequence of shipping experience were minimized between cages. Briefly, the first box was opened and one mouse was added to each cage in the following order, Type 2, Type 3, Pet (Step 1; 9 cages). A second mouse was then added to the cages containing groups of 5 and groups of 8 (Step 2; 6

cages). A third mouse was then added to the cages containing groups of 8 (Step 3; 3 cages). Steps 1 to 3 were then repeated, moving onto the second box of animals when the first was empty and then, steps 1 to 2 were repeated. Cage position on the rack (height and side) was counterbalanced by sex, strain, and group size between batches.

d) Ear-Tattooing of subjects

All mice were individually marked by ear tattoo. Animals of batch 1 were tattooed on the day of arrival immediately after allocation to cages. However, due to the death of one animal during restraint, mice of batches 2 to 6 were tattooed on the day following arrival to permit for recovery from transport and for acclimatization to re-housing. The same two experimenters (JDB and EM) tattooed the animals across all batches. Briefly, each animal was restrained by the scruff (JDB) and then tattooed (EM) in one of eight pre-defined ear locations.

Supplementary Methods 2: Open Field

The open field test was originally developed by Hall[1], as a means of assessing emotionality in rats. Since then, many variations of this test have been produced, including differences in light intensity, time of testing, size of the apparatus, and measurement of outcome variables, to name a few. Thus, the term “open field test” has virtually no meaning without explicit specification of what construct is being measured[2–4].

The open field remains a popular apparatus in rodent research, and in particular, has been used to identify and validate behavioural differences related to anxiety in both rats and mice[5,6]. More specifically, longitudinal assessment of the pattern of locomotor behaviour in the open field across repeated exposures has been demonstrated to provide information about how groups of animals cope with the presentation of a stressor and how the HPA axis differentially operates between groups of animals[7].

In the present experiment, four open field arenas made of polycarbonate with dimensions 45 x 45 x 45 cm[3] were used to test mice in squads of four. Behaviour in the arena was digitally recorded (960P, 30 fps), across four days between 10:00 to 13:00. Overhead lighting was maintained at 40 lux in the centre of each field, and at 30 lux near the walls based on the recommendations of Martin-Arenas and Pintado[8]. Mice from four cages were tested per squad in a pseudo-random manner by two experimenters (EM and JDB), counterbalanced between batches for strain, sex, and treatment. Nine squads of animals (n = 36 mice, 4 per squad) were tested from batches 1 to 4 and seven squads (n = 27 mice, 3 per squad) from batches 5 to 6. Data from four squads (12 animals) on day 1 of testing were lost from batch 2 because of digital recorder failure.

Briefly, the cages which contained the four animals to be tested were brought to the testing room and the doors to the housing room were closed. The overhead lights were turned on and the four animals were removed from the cages. Each animal was placed into a single arena and allowed to explore for ten minutes. The animals were then weighed, replaced in the

home-cage, and returned to the housing room. The arenas were then cleaned with 70% ethanol and the next squad was tested.

Digital recordings were processed in Noldus EthoVision XT (version 11.5) by JDB. In EthoVision, the unit of distance was centimetres (cm) and the unit of time was seconds (s). The floor of the arena was divided into sixteen equal squares (11.25 x 11.25 cm^2), with the four squares in the middle representing the centre of the arena and the remaining squares the periphery. The detection settings for tracking were selected so that both the percentage of samples in which the subject was not found and the percentage of samples skipped were less than 1% per trial. To ensure accuracy, a human observer verified the tracking of the software live as the recordings were analysed. Furthermore, each trial was edited within EthoVision such that each point was accurately scored and issues associated with automated tracking were eliminated[9]. The outcome variables of interest across the four days of testing were: 1) distance travelled, and 2) time spent in the centre.

Supplementary Methods 3: Guessing Task

In the present study, we used the same apparatus as described by Novak and colleagues[10]. The apparatus consisted of a box made of polycarbonate (20 x 50 cm^2) with a start box (10 x 10 cm^2) and two goal compartments (10 x 20 cm^2) each containing a goal-pot (Supplementary Figure 3). Subjects from each batch were tested in two cohorts in a pseudo-random manner by the same two experimenters (JDB, EM), counterbalanced for sex, strain, and treatment under red light in the dark phase. The timeline for a single cohort is shown below (Supplementary Figure 4).

Food Restriction: On the day following the completion of the open field test, cages were changed. The first squad of animals was fed 85% of their *ad libitum* daily consumption (based on the previous week's intake) for the next seven days. Of the 85% *ad libitum* ration, approximately 3 g of Bio-Serv™ dustless chocolate precision pellets (20 mg) were were placed in a goal-pot and put in the home cage on the first three days of food restriction, to reduce neophobia to the reward and associate the goal-pot with the presence of reward. All animals in the cage were weighed daily to ensure that body weight did not fall below 85% of the pre-restriction weight. If an animal's weight dropped below 85%, it was placed in a separate cage and fed *ad libitum* for 30 minutes.

Habituation: Habituation to the apparatus occurred across three days. On the second day of food restriction, the focal test animal and one randomly selected cage-mate were placed into the apparatus. Both goal-pots were present and six pellets were placed in each goal-pot as well as on the floor. After ten minutes, both animals were removed, the entire cage weighed, and then fed. On the third day, the same procedure was repeated but with only the focal animal. The procedure for the fourth day was the same as on day three, with the exception that there were two sessions of habituation (morning and afternoon) for five minutes. Each cage was fed only after the second session.

Shaping: On the fifth day of food restriction, each mouse received 12 training trials across two sessions (morning and afternoon), in which both goal-pots were baited. In all trials, both goal-pots contained five inaccessible pellets at the bottom which was covered with wire mesh and served as a control for odour cues. Between mice, but not between trials, the apparatus was cleaned with a 70% ethanol solution. As soon as the mouse entered one compartment, access to the other compartment was blocked by closing the guillotine door. If the mouse chose the same side three times in succession, that side was closed in the following trial to avoid shaping the mouse to one side. A trial was completed when the animal's head (nose) was above the goal-pot, after which the animal was left to eat the reward. The mouse was then returned to the start box and the next trial begun. The cage was fed only after the second session.

Testing: The test phase consisted of 100 trials conducted over a maximum of three sessions; although all but 3 animals completed 100 trials across 2 days. For each trial, the start box door was opened and when the animal selected a goal-pot, the other compartment was closed. The animal was left to eat the pellet (if the choice was correct) and then returned to the start box. Each session was terminated after 30 minutes or as soon as the mouse started showing off-task behaviour[11].

For each trial, only one goal-pot was baited, with a probability equalling the proportion of responses to the other side in the previous 20 trials. This randomization procedure was used to eliminate side biases which may confound the experimental paradigm and was determined by a custom written computer program[12]. In trials 1 to 19, side bias was calculated from all previous trials. Although reward side is unpredictable, choosing each side equally often will maximize the number of rewards. The mouse can do so by producing either a random or patterned sequence of responses. Patterned sequences (which show high sequential dependence) can be apparent as either series of repetitions or alternations or more complex sequential patterns, and indicate recurrent perseveration[13,14].

Perseveration score (logit P) was used as the primary outcome measure of recurrent perseveration. The score was calculated using 3rd order Markov chain analysis[15], which describes the probability of a behaviour occurring as a function of previous behaviour (where the 3rd order considers the three previous behavioural responses) and provides a method to assess sequential independence while controlling for side bias. These analyses were performed using a custom written computer program which calculated the observed and expected probabilities of each choice regarding each of the 16 possible configurations (see below) of the three previous choices. Then the sum chi-square was calculated from the observed and expected values. The probability of each sum chi-square (p) indicated the probability of sequential independence of the observed sequence. Therefore, recurrent perseveration was calculated by (1-p), where 1 represents a completely perseverant sequence. These data were logit transformed (logit P) to yield an unbounded variable suitable for regression analyses.

Additionally, the distribution of tetragrams (sequences of four trials) was examined by dividing each response sequence consisting of 100 trials per mouse into 97 overlapping tetragrams. Sixteen configurations of tetragrams were possible, of which two were pure repetitions and two were pure alternations. A random search strategy should be characterized by an equal distribution of all possible configurations (97/16 = 6), whereas perseverative behaviour should result in sequences characterized by higher rates of alternations or repetitions[16].

Supplementary Table 1: Comparison of space guidelines for non-breeding mice: a) used in research by jurisdiction and b) mice housed as pets compared to those housed for research in Switzerland.

a

Reference	Body Weight (g)	Floor Area (cm^2)	Height (cm)	Floor Area/Mouse (cm^2)
EC Directive 2010/63/EU (European Union, 2010)	< 20<br>20-25<br>25-30<br>> 30	330	12	60<br>70<br>80<br>100
Animal Welfare Ordinance, 455.1 (Switzerland, 2011)	< 20<br>20-30<br>> 30	330	12	60<br>80<br>100
The Guide, 8th Edition (USA, 2011)	< 10<br>Up to 15<br>Up to 25<br>> 25	Not provided	12.7	38.7<br>51.6<br>77.4<br>≥ 96.7
Canadian Council on Animal Care (Canada, 1993)	< 20<br>> 20	Not provided	13<br>15	65<br>100

Note: The figures quoted above reflect the minimum standards for each jurisdiction

b

	# of Animals	Floor Area (cm^2)	Floor Area/Mouse (cm^2)	Every additional mouse (cm)
**Laboratory**	2	330	60-100	N/A
**Pets**	2	1800	900	500

Note: The figures quoted above reflect the minimum standards of the Swiss Animal Welfare Ordinance (455.1)

Supplementary Table 2. Distribution of the total number of subjects delivered across batches.

Batch	Delivery Date	C57		BALB	
		Males	Females	Males	Females
1	07/08/2014	47	48	48	48
2	11/09/2014	48	48	48	47
3	16/10/2014	48	47	48	48
4	22/01/2015	48	48	48	48
5	26/02/2015	48	48	-	47
6	02/04/2015	48	48	-	48
	**Total**	287	287	192	286
		574		478	

Supplementary Table 3. Ethogram for the recording of stereotypic behaviour.
Behaviour patterns were considered stereotypic if the same movement sequence was repeated continuously for at least 3 s (bar-mouthing) or at least three times in a row without pauses longer than 3 s between bouts (circling, cage-top twirling, back-flipping, route-tracing).

Category	Name	Definition
General activity	Active	The animal is active throughout the 15s interval
	Inactive	Sitting or lying motionless throughout the 15s interval, occasionally interrupted by brief single twitches lasting no longer than 5 s.
Stereotypic behaviour	Bar-mouthing	The mouse holds the cage bar in its diastema and makes a series of sham-biting or open mouth movements along the bar.
	Circling	Repetitive tracing of a circular path either on the cage lid or floor.
	Cage-top twirling	Spinning around the longitudinal body axis while hanging on the cage lid with the forepaws.
	Back-flipping	Backward flip from one cage wall towards the opposite cage wall.
	Route-tracing	Moving along an invariant route on the cage lid or floor.

Supplementary Table 4. Distribution of stereotypic behaviour by strain and sex.
Numbers outside brackets represent the number of cages (focal animal/cage). The proportion of the total number of cages is expressed within brackets. For stereotypy types, proportions are calculated from the total number of animals displaying stereotypic behaviour.

Strain	Sex	No Stereotypy	Stereotypic	Bar-mouthing	Route-tracing	Circling	Cage-top twirling	Back-flipping	Combined
BALB	Males	3 (18%)	14 (82%)	11 (78%)	2 (14%)	0 (0%)	3 (21%)	0 (0%)	2 (14%)
	Females	14 (26%)	40 (74%)	37 (93%)	2 (5%)	2 (5%)	0 (0%)	1 (3%)	2 (5%)
	Strain Total	***17 (24%)***	***54 (76%)***	***48 (89%)***	***4 (7%)***	***2 (4%)***	***3 (6%)***	***1 (2%)***	***4 (7%)***
C57	Males	49 (93%)	4 (7%)	0 (0%)	4 (100%)	0 (0%)	0 (0%)	0 (0%)	0 (0%)
	Females	46 (85%)	8 (15%)	1 (13%)	6 (75%)	1 (13%)	0 (0%)	0 (0%)	0 (0%)
	Strain Total	***95 (89%)***	***12 (11%)***	***1 (8%)***	***10 (83%)***	***1 (8%)***	***0 (0%)***	***0 (0%)***	***0 (0%)***
	**Total**	**112 (63%)**	**66 (37%)**	**49 (74%)**	**14 (21%)**	**3 (5%)**	**3 (5%)**	**1 (2%)**	**2 (3%)**

Combined stereotypies = bar-mouthing and back-flipping (x1), bar-mouthing and route-tracing (x2), and bar-mouthing and twirling (x1).

Supplementary Table 5. Mean proportion of intervals (± 95% CI) of observed stereotypic behaviour by type.
Average values are calculated within stereotypy type, while the estimate of stereotypic behaviour is calculated between stereotypy type.

Strain	Sex	Bar-mouthing	Route-tracing	Circling	Cage-top twirling	Back-flipping	Combined	**Total**
BALB	Males	15 ± 19	1 ± 6	0 ± 0	6 ± 12	0 ± 0	15 ± 53	19 ± 21
	Females	21 ± 13	0 ± 2	1 ± 3	0 ± 0	0 ± 0	18 ± 66	19 ± 14
C57	Males	0 ± 0	3 ± 15	0 ± 0	0 ± 0	0 ± 0	0 ± 0	2 ± 12
	Females	0 ± 2	20 ± 9	6 ± 16	0 ± 0	0 ± 0	0 ± 0	15 ± 25

Supplementary Table 6. Distribution of missing data (%) for BALB males that reached early termination criteria. Numbers outside brackets represent the number of cages removed due to attrition from the experiment and the proportion of the total number of cages, n=4, is expressed within brackets.

		Type 2	Type 3	Pet
**Floor Area (cm^2)**		**370**	**820**	**2400**
**Group Size**	**3**	0 (0%)	2 (50%)	1 (25%)
	**5**	1 (25%)	3 (75%)	2 (50%)
	**8**	3 (75%)	3 (75%)	4 (100%)

Supplementary Table 7. Summary of outcome variables relating to sex and age by strain

**Strain**	**C57**			**BALB**		
**Outcome Measure**	***Sex***	***Age/Time***	***Sex x Age/Time***	***Sex***	***Age/Time***	***Sex x Age/Time***
Food intake	*Males ate more* $F_{1,99} = 219.47$	*Increased with age* $F_{6,346} = 207.07$	*Males ate more across all ages* $F_{6,346} = 24.27$	*Males ate more* $F_{1,84} = 190.02$	*Increased with age* $F_{6,223} = 54.72$	*Males ate more across all ages* $F_{6,223} = 3.98$
Water intake	*Males drank more* $F_{1,100} = 111.58$	*Increased with age* $F_{6,311} = 75.84$	*n.d.* $F_{6,311} = 1.91$	*Males drank more* $F_{1,83} = 113.72$	*Increased with age* $F_{6,260} = 46.50$	*Males drank more across all ages* $F_{6,260} = 3.34$
Body weight	*Males weighed more* $F_{1,635} = 783.22$	*Increased with age* $F_{7,3566} = 2613.18$	*Males weighed more across all ages* $F_{7,3566} = 133.17$	*Males weighed more* $F_{1,512} = 572.46$	*Increased with age* $F_{7,3021} = 2291.59$	*Males weighed more across all ages* $F_{7,3021 = 89.29}$
Glucocorticoid metabolite concentrations	*Females higher than males* $F_{1,95} = 12.01$	*Decreased between weeks 5, 7 and 10* $F_{3,216} = 19.45$	*Females lower in week 5, and higher in week 7 and 10* $F_{3,216} = 11.59$	*Females higher than males* $F_{1,124} = 378.96$	*Decreased between week 5 and 7* $F_{3,230} = 4.59$	*Females higher across all time points* $F_{3,230} = 17.46$
Open field: distance travelled	*n.d.* $F_{1,113} = 1.16$	*Decreased across time* $F_{3,278} = 77.06$	*n.d.* $F_{3,278} = 1.27$	*Males higher than females* $F_{1,91} = 18.72$	*Increased across time* $F_{3,234} = 9.51$	*Males higher across time* $F_{3,234} = 2.75$
Open field: time in centre	*n.d.* $F_{1,113} = 0.01$	*Decreased across time* $F_{3,266} = 61.27$	*n.d.* $F_{3,266} = 0.08$	*Females higher than males* $F_{1,90} = 9.37$	*Decreased across time* $F_{3,228} = 14.05$	*n.d.* $F_{3,228} = 0.22$
Guessing task: perseveration score	*n.d.* $F_{1,98} = 0.40$	-	-	*n.d.* $F_{1,69} = 0.81$	-	-
Guessing task: distribution of tetragrams	*n.d.* $F_{15,1643} = 6.40$	-	-	*n.d.* $F_{15,1179} = 21.02$	-	-
Home-cage behaviour: activity	*n.d.* $U = 1.42$	-	-	*n.d.* $U = 0.41$	-	-

n.d = no observed differences

Supplementary Table 8. Distribution of outcome data, collected and missing, by treatment, strain and sex.
As the treatments in this experiment were contingent upon the maintenance of a constant group size throughout the duration of the experiment, complete data was not available for some of our outcome measures. However, for outcomes with repeated measurement such as body weight, data at available time points were used to inform parameter estimates of values when possible.

	Strain	BALB																	
	Sex	Males									Females								
	Cage Type	Type 2			Type 3			Pet			Type 2			Type 3			Pet		
	Group Size	3	5	8	3	5	8	3	5	8	3	5	8	3	5	8	3	5	8
Collected (n)	Food Intake (x7 Weeks)	28	28	25	26	28	26	28	28	25	42	35	36	42	42	42	40	42	42
	Water Intake (x7 Weeks)	28	28	25	26	28	26	28	28	25	42	35	36	42	42	42	40	42	42
	Body Mass (x8 Weeks)	96	160	232	90	160	240	96	160	233	144	200	336	144	240	384	138	240	384
	Corticosterone Metabolites	16	16	12	13	13	13	15	14	13	23	20	20	24	24	24	24	24	23
	Open-Field (x4 Days)	16	16	16	12	16	14	16	16	10	24	20	20	23	23	23	24	24	24
	Guessing Task	4	4	1	3	2	1	3	4	0	6	5	5	6	5	6	6	6	6
	Stereotypic Behaviour	4	4	1	2	1	1	3	1	0	6	5	5	6	6	6	6	6	6
Missing (n)	Food Intake (x7 Weeks)	0	0	3	2	0	2	0	0	3	0	7	6	0	0	0	2	0	0
	Water Intake (x7 Weeks)	0	0	3	2	0	2	0	0	3	0	7	6	0	0	0	2	0	0
	Body Mass (x8 Weeks)	0	0	24	6	0	24	0	0	24	0	40	48	0	0	0	6	0	0
	Corticosterone Metabolites	0	0	4	3	3	3	1	2	3	1	4	4	0	0	0	0	0	1
	Open-Field (x4 Days)	0	0	0	4	0	2	0	0	6	0	0	0	0	0	0	0	0	0
	Guessing Task	0	0	3	1	2	3	1	0	4	0	1	1	0	1	0	0	0	0
	Stereotypic Behaviour	0	0	3	2	3	3	1	3	4	0	1	1	0	0	0	0	0	0

	Strain	C57																	
	Sex	Males									Females								
	Cage Type	Type 2			Type 3			Pet			Type 2			Type 3			Pet		
	Group Size	3	5	8	3	5	8	3	5	8	3	5	8	3	5	8	3	5	8
Collected (n)	Food Intake (x7 Weeks)	42	42	35	42	42	41	42	42	42	42	42	42	42	42	42	42	42	33
	Water Intake (x7 Weeks)	42	42	35	41	41	41	41	42	42	42	42	42	41	41	41	42	42	33
	Body Mass (x8 Weeks)	144	240	320	144	240	384	144	240	384	144	240	384	144	240	384	144	240	304
	Corticosterone Metabolites	24	24	20	24	24	24	24	24	24	24	24	24	24	24	24	24	24	20
	Open-Field (x4 Days)	24	23	20	24	24	24	24	23	24	23	23	24	24	24	23	23	23	20
	Guessing Task	6	6	5	6	5	6	6	6	6	5	6	6	5	6	6	6	6	5
	Stereotypic Behaviour	6	6	5	6	6	6	6	6	6	6	6	6	6	6	6	6	6	5
Missing (n)	Food Intake (x7 Weeks)	0	0	7	0	0	1	0	0	0	0	0	0	0	0	0	0	0	9
	Water Intake (x7 Weeks)	0	0	7	1	1	1	1	0	0	0	0	0	1	1	1	0	0	9
	Body Mass (x8 Weeks)	0	0	64	0	0	0	0	0	0	0	0	0	0	0	0	0	0	80
	Corticosterone Metabolites	0	0	4	0	0	0	0	0	0	0	0	0	0	0	0	0	0	4
	Open-Field (x4 Days)	0	0	0	0	0	0	0	0	0	0	0	0	0	0	0	0	0	0
	Guessing Task	0	0	1	0	1	0	0	0	0	1	0	0	1	0	0	0	0	1
	Stereotypic Behaviour	0	0	1	0	0	0	0	0	0	0	0	0	0	0	0	0	0	1

Supplementary Figure 1: Images and dimensions (L x W x H, cm) of the cages used in this experiment.

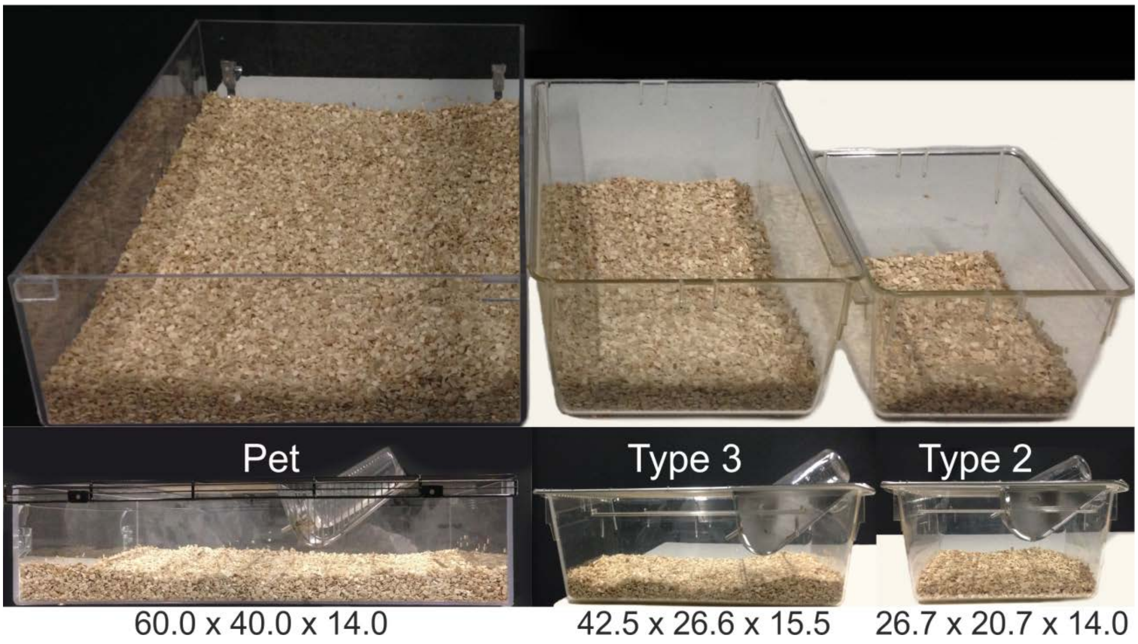

Supplementary Figure 2. Example of layout of housing and testing rooms for two batches

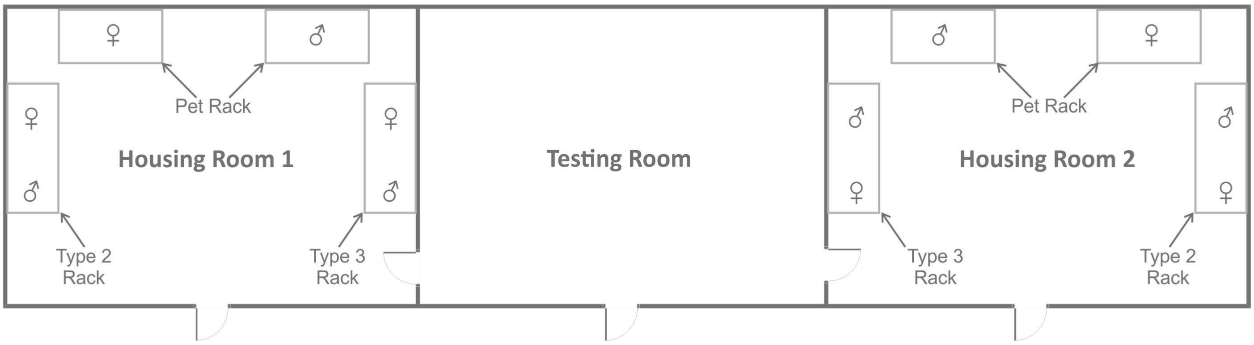

Supplementary Figure 3. Guessing task apparatus

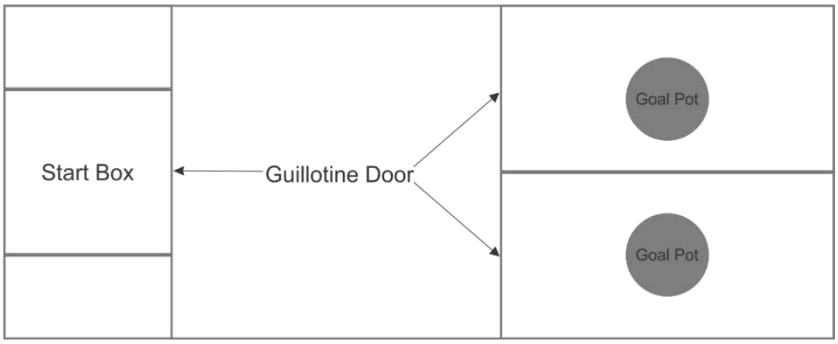

Supplementary Figure 4. Testing timeline for a single cohort in the guessing task

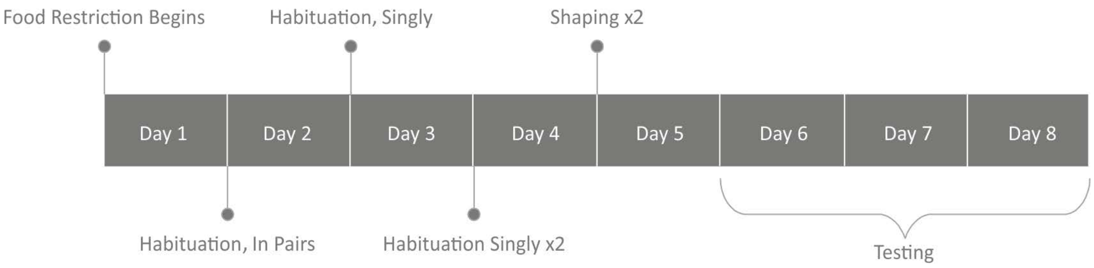

Supplementary Figure 5. Score sheet used for assessment of general body condition, aggression and evaluation of humane end-points

Daily score sheet for general condition:

- Persons responsible for observation of the animals will be trained by their supervisors to identify signs of impaired health and well-being exhibited by the species used, as well as the humane endpoints defined in the experimental licences.
- Each animal will be monitored for Appearance, Activity, and Eyes/Nose daily; and assigned a score for each parameter as described:

Parameter	Grade	Description	Score
Appearance	Normal	Normal posture, shiny and well-groomed hair coat	3
	Mild	Normal posture, unkempt hair coat and dull fur	2
	Moderate	Hunching, markedly unkempt hair coat	1
	Severe	Scored as moderate (1) for more than 12 hours; Hair loss	0
Activity	Normal	Active; interactive with environment	3
	Mild	Slight decrease in activity; less interactive	2
	Moderate	Pronounced decrease in activity	1
	Severe	Scored as moderate (1) for more than 12 hours; Ataxia, limb paralysis, no locomotion after moderate stimulation	0
Eyes and Nose	Normal	Bright eyes	3
	Mild	Eyes squinted, no discharge	2
	Moderate	Eyes closed, no discharge	1
	Severe	Scored as moderate (1) for more than 12 hours; discharge	0

- If an animal receives a score of 3 in all parameters, it is given an overall score of 3 and the necessary action is taken.
- If an animal receives a score of less than 3 on any single parameter, this score represents the overall score and the necessary action is taken (e.g. Animal scored 1 in appearance, 2 in Activity, and 3 in Eyes and Nose; is given an overall score of 1).

Grade	Action	Overall Score
Normal	No action; monitor daily	3
Mild	Monitor two times per day, notify primary investigator	2
Moderate	Monitor three times per day, notify primary investigator	1
Severe	Termination criteria – euthanize cage	0

Daily score sheet for aggression:

- Each animal will be monitored for aggression and assigned a score as described below and the necessary action will be taken:

Parameter	Grade	Description	Score
Aggression	Normal	Occasional fighting, mice are incurring no wounds	3
	Mild	Occasional fighting, and a mouse may have a single wound on the tail or body	2
	Moderate	Fighting has increased, and a mouse has incurred multiple wounds on tail or body	1
	Severe	Fighting and number of wounds have progressively increased over 2 days; Mouse has a gaping and open wound	0

Grade	Action	Score
Normal	No action; monitor daily	3
Mild	Monitor two times per day, notify primary investigator	2
Moderate	Monitor three times per day, notify primary investigator	1
Severe	Termination criteria – euthanize cage	0

Supplementary Figure 6. Food intake by sex, age and strain

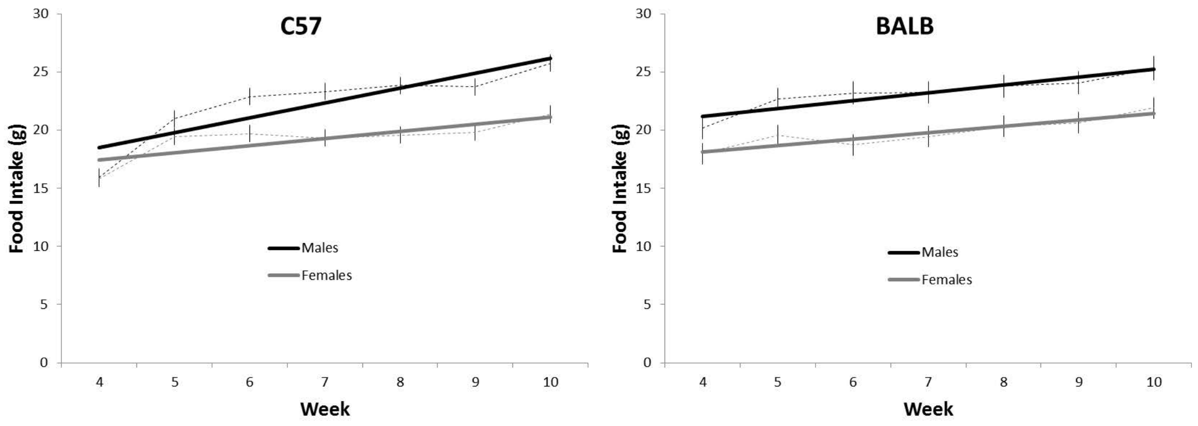

Supplementary Figure 7. Water intake by sex, age and strain

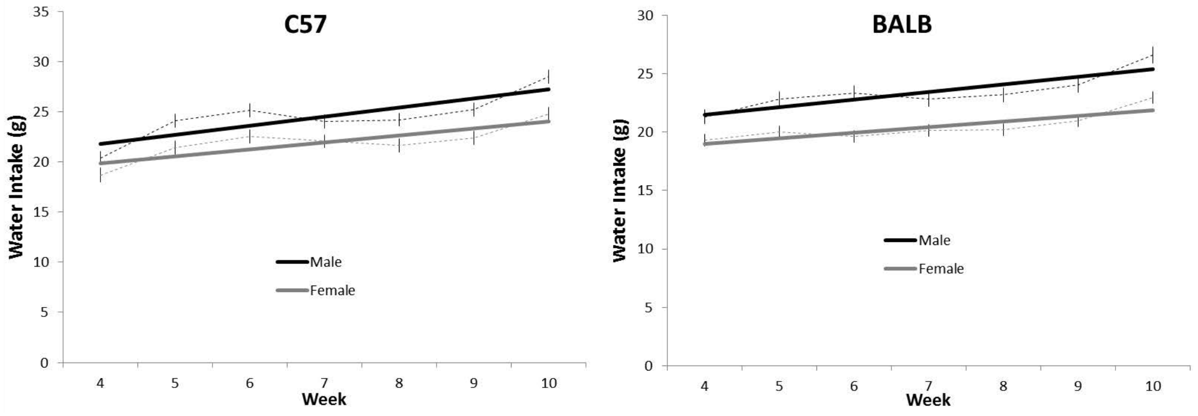

Supplementary Figure 8. Interaction between group size and age with respect to body mass in C57 mice

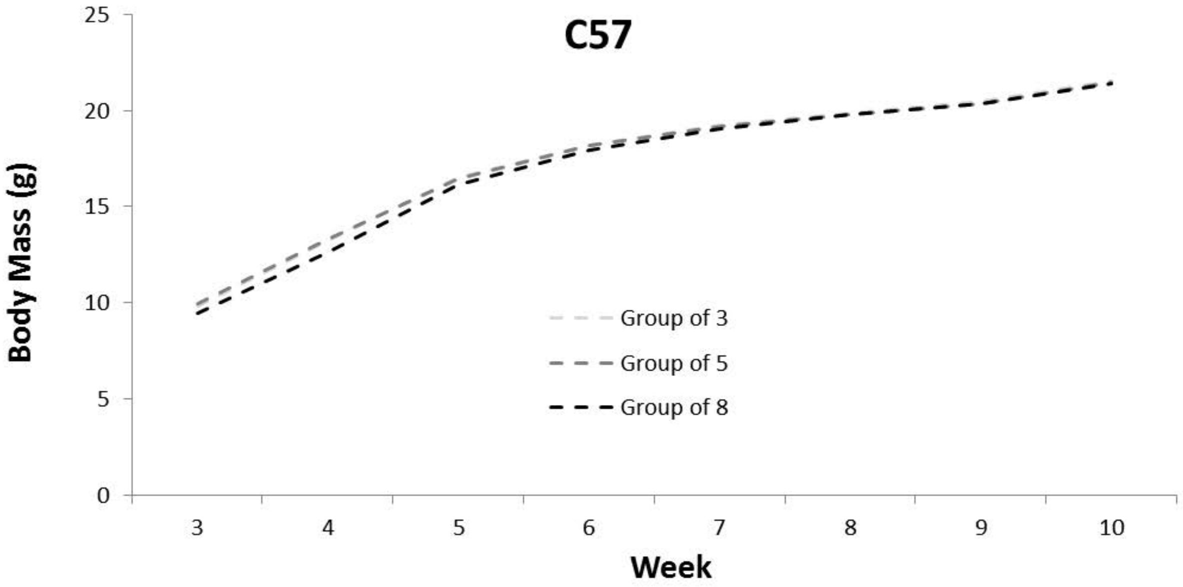

Supplementary Figure 9. Interaction between floor area and age with respect to body mass

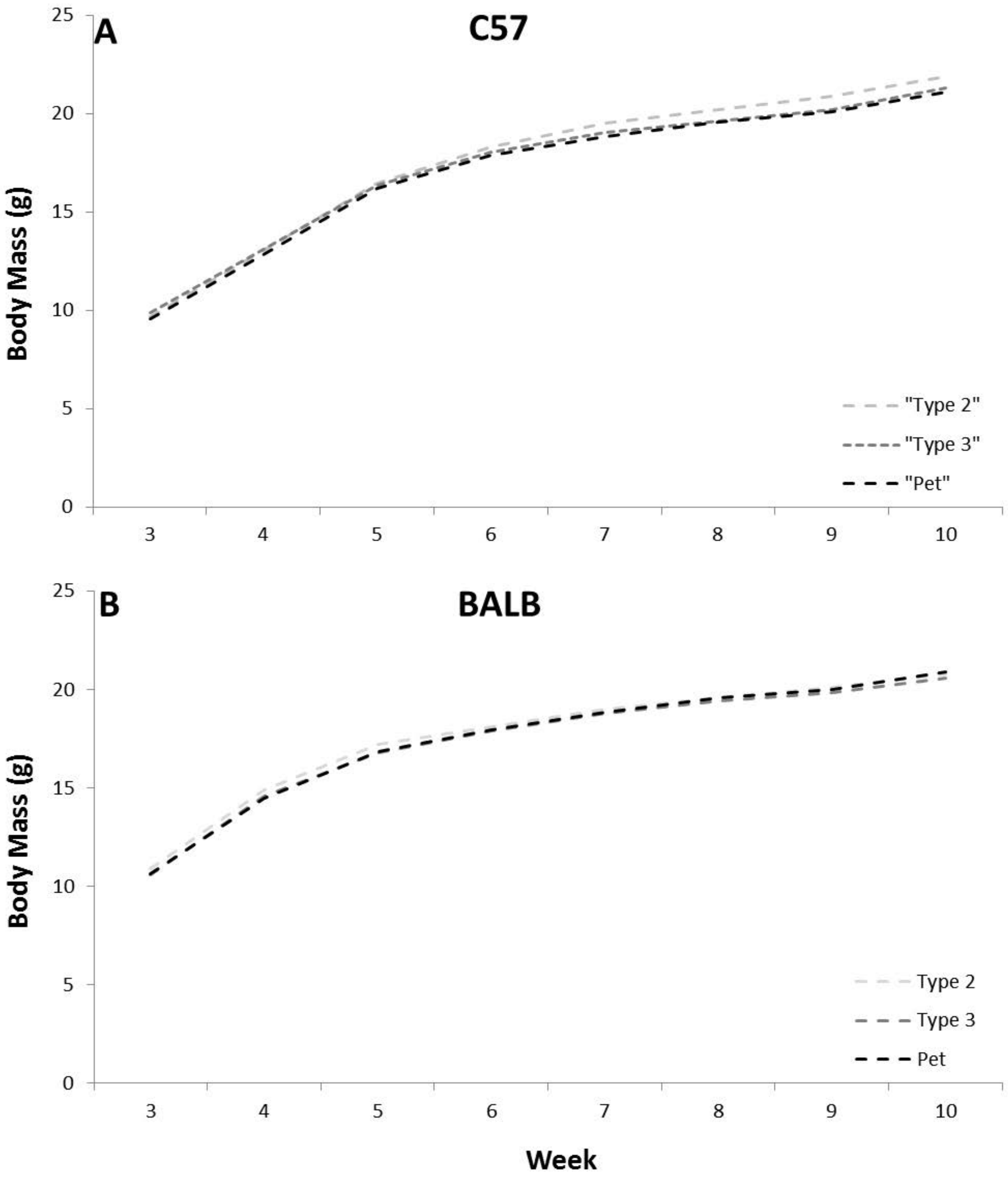

Supplementary Figure 10. Body mass by sex, age and strain

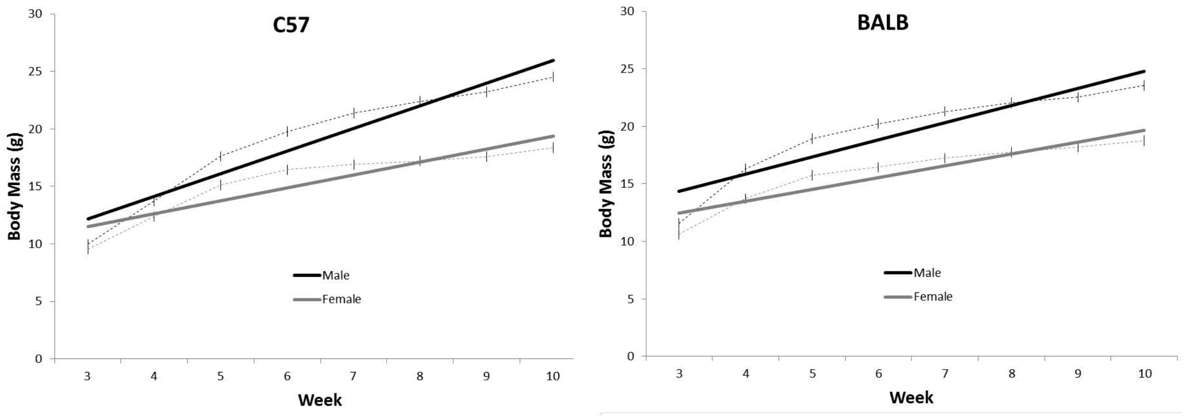

Supplementary Figure 11. Glucocorticoid metabolite concentrations (Estimated Marginal Means ± 95% CI) by: a) increased group size, controlling for floor area, b) increased group size, space allocation constant, c) increased floor area, controlling for group size and d) lab vs. pet standards.

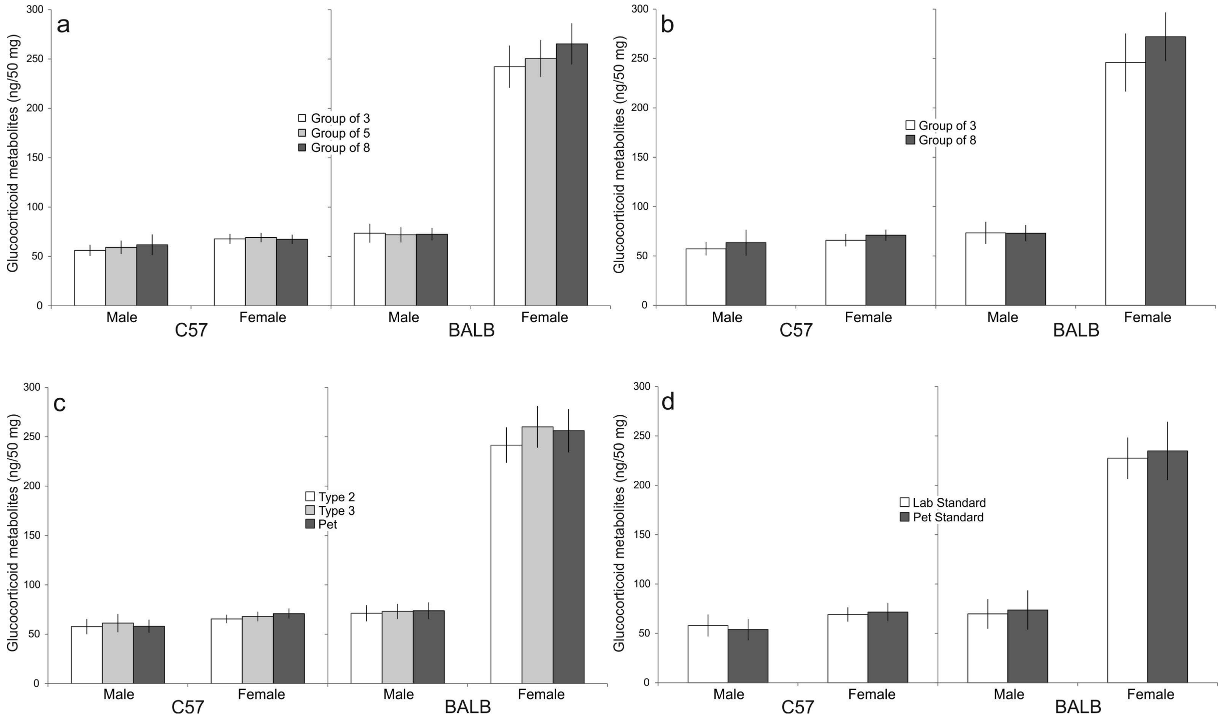

Supplementary Figure 12. Glucocorticoid metabolite concentrations by sex, week and strain

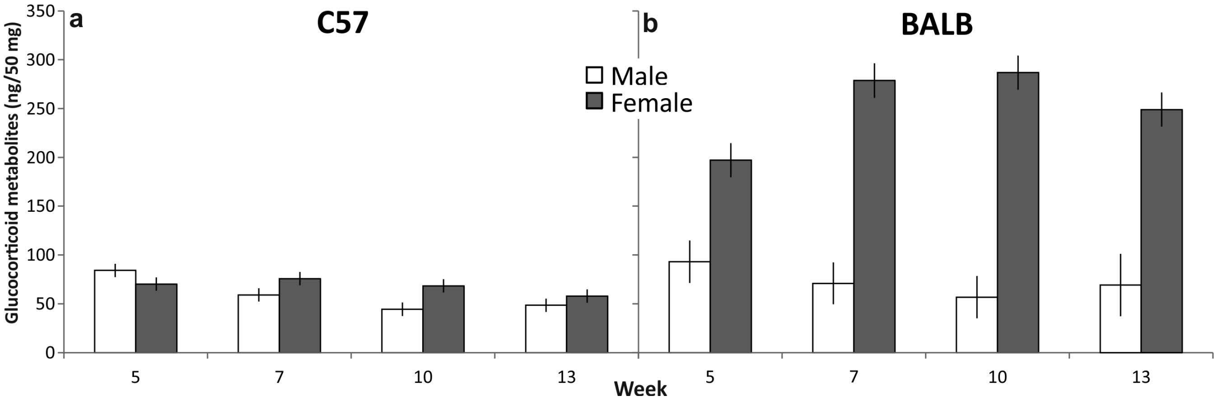

Supplementary Figure 13. Distance travelled in the open field (Estimated Marginal Means ± 95% CI) by: a) increased group size, controlling for floor area, b) increased group size, space allocation constant, c) increased floor area, controlling for group size and d) lab vs. pet standards.

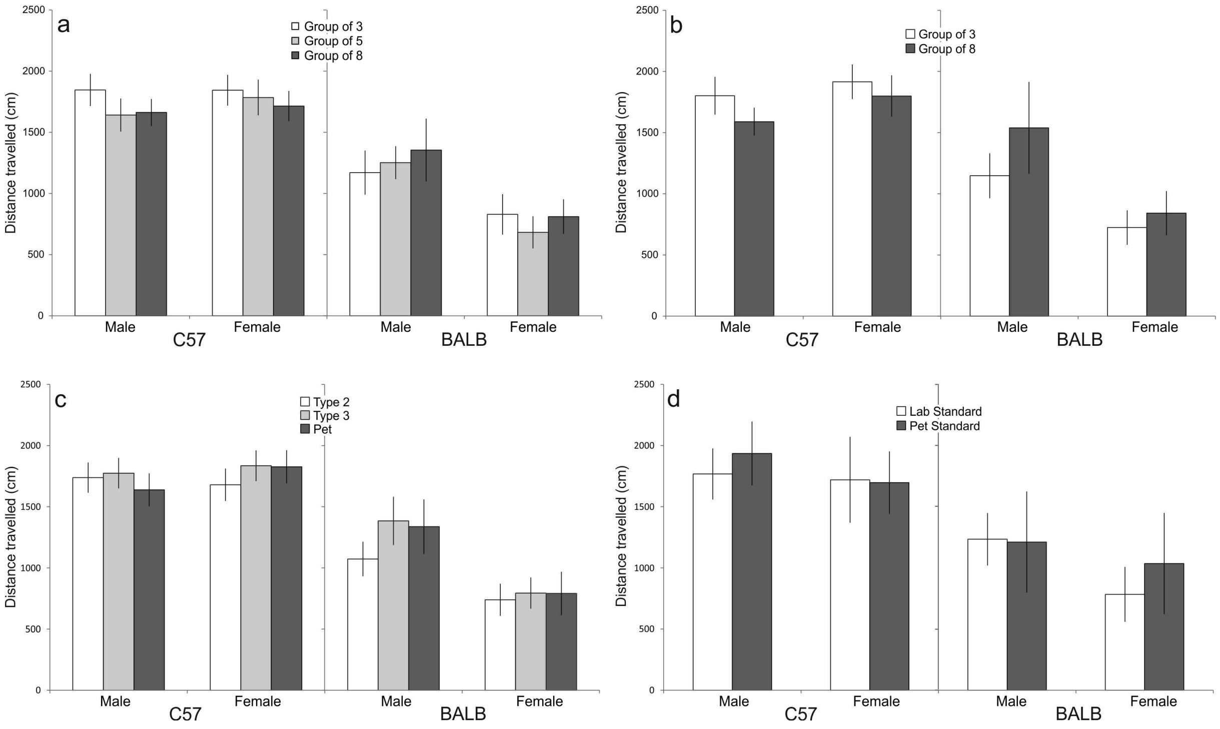

Supplementary Figure 14. Time spent in the centre of the open field (Estimated Marginal Means ± 95% CI) by: a) increased group size, controlling for floor area, b) increased group size, space allocation constant, c) increased floor area, controlling for group size and d) lab vs. pet standards.

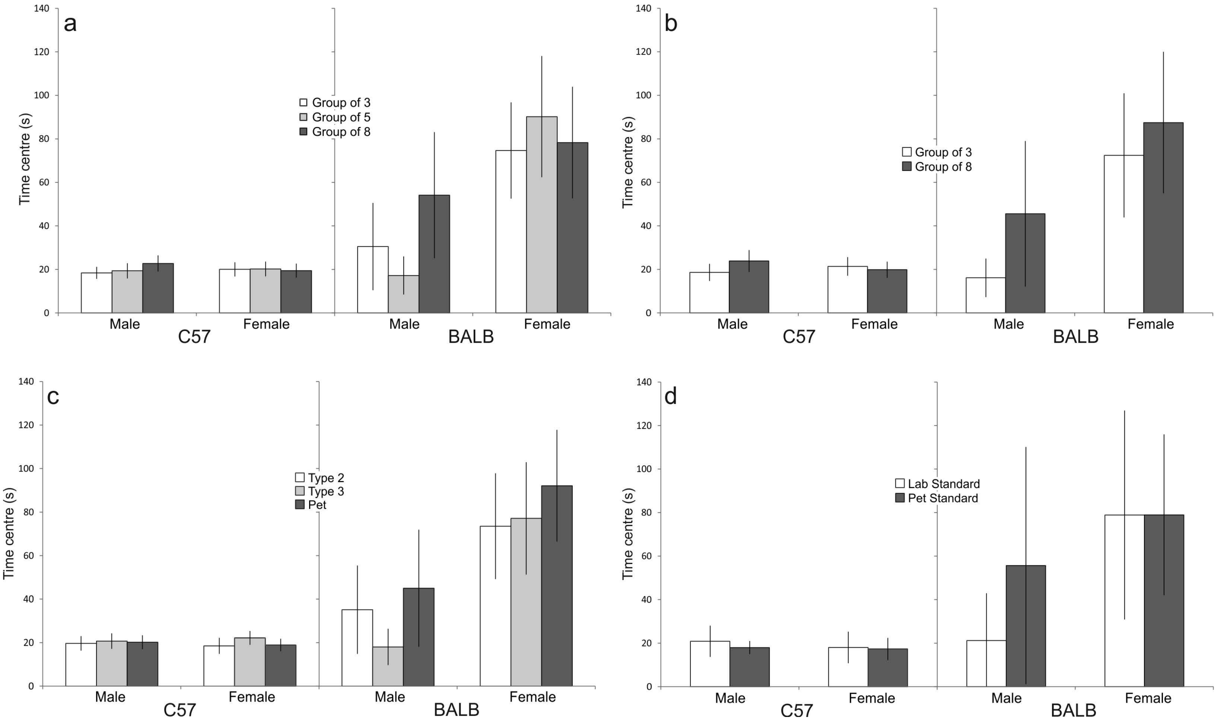

Supplementary Figure 15. Distance travelled and time spent in the centre of the open field by sex and week in C57 mice.

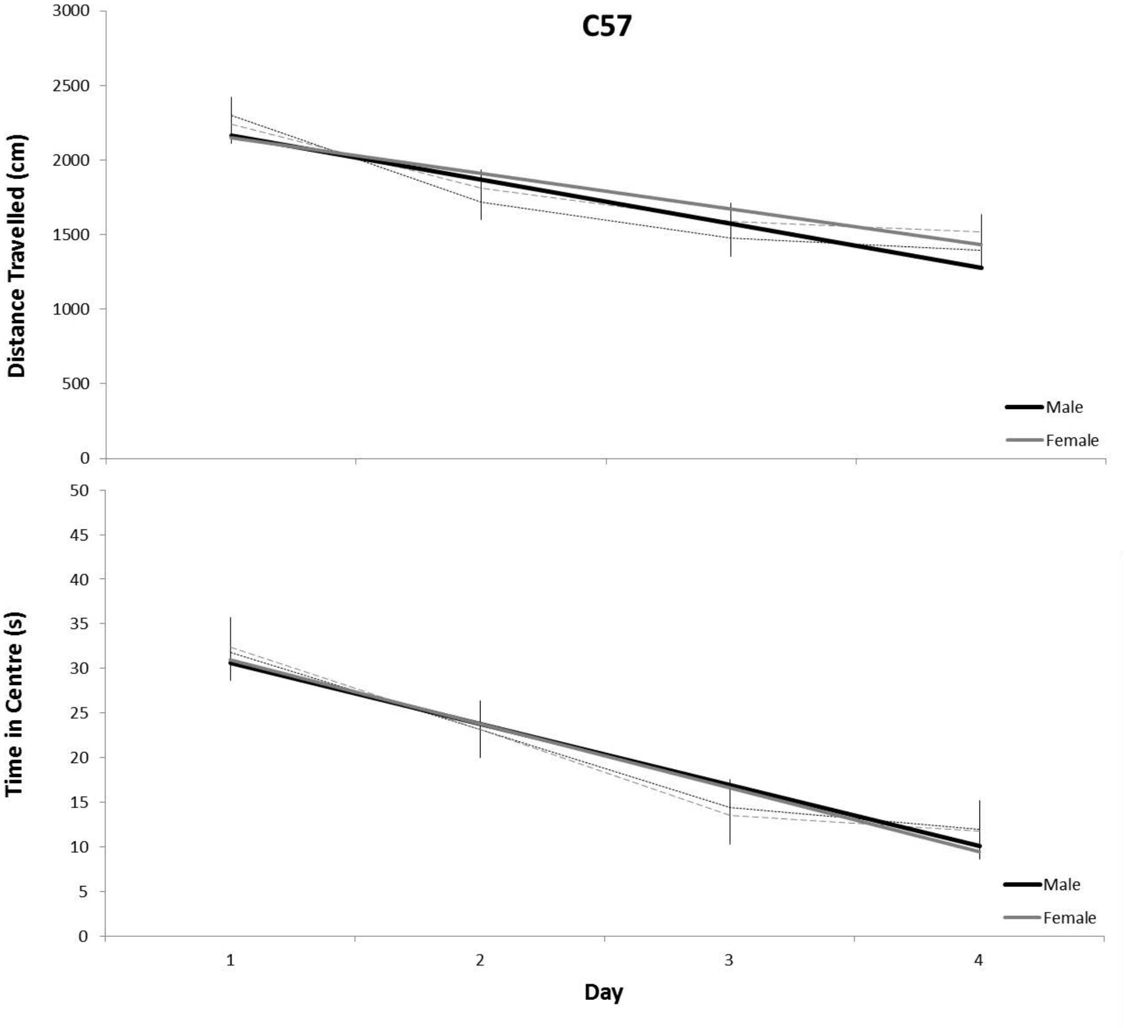

Supplementary Figure 16. Distance travelled and time spent in the centre of the open field by sex and week in BALB mice.

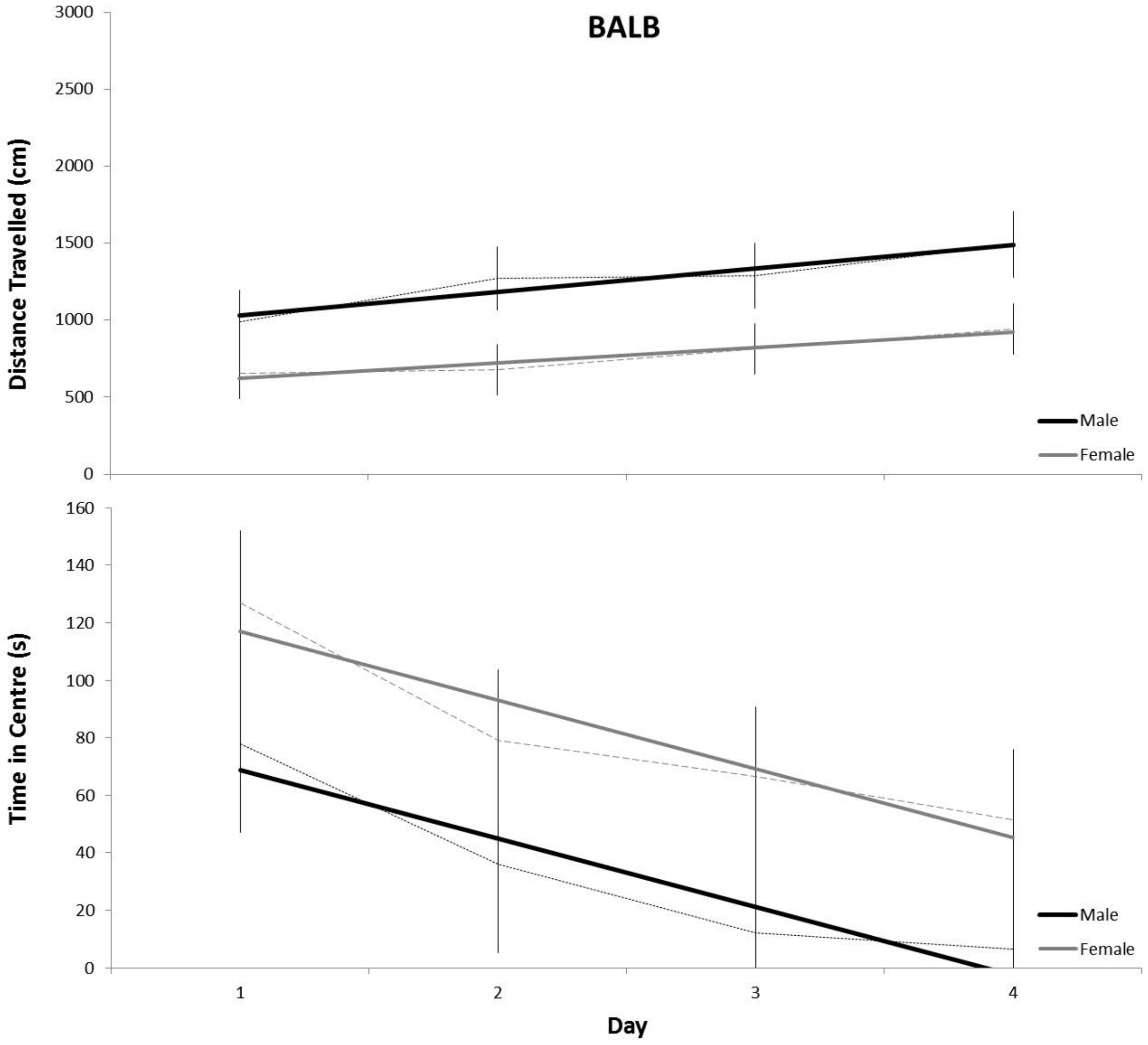

Supplementary Figure 17. Perseveration score (Estimated Marginal Means ± 95% CI) by: a) increased group size, controlling for floor area, b) increased group size, space allocation constant, b) increased floor area, controlling for group size and d) lab vs. pet standards.

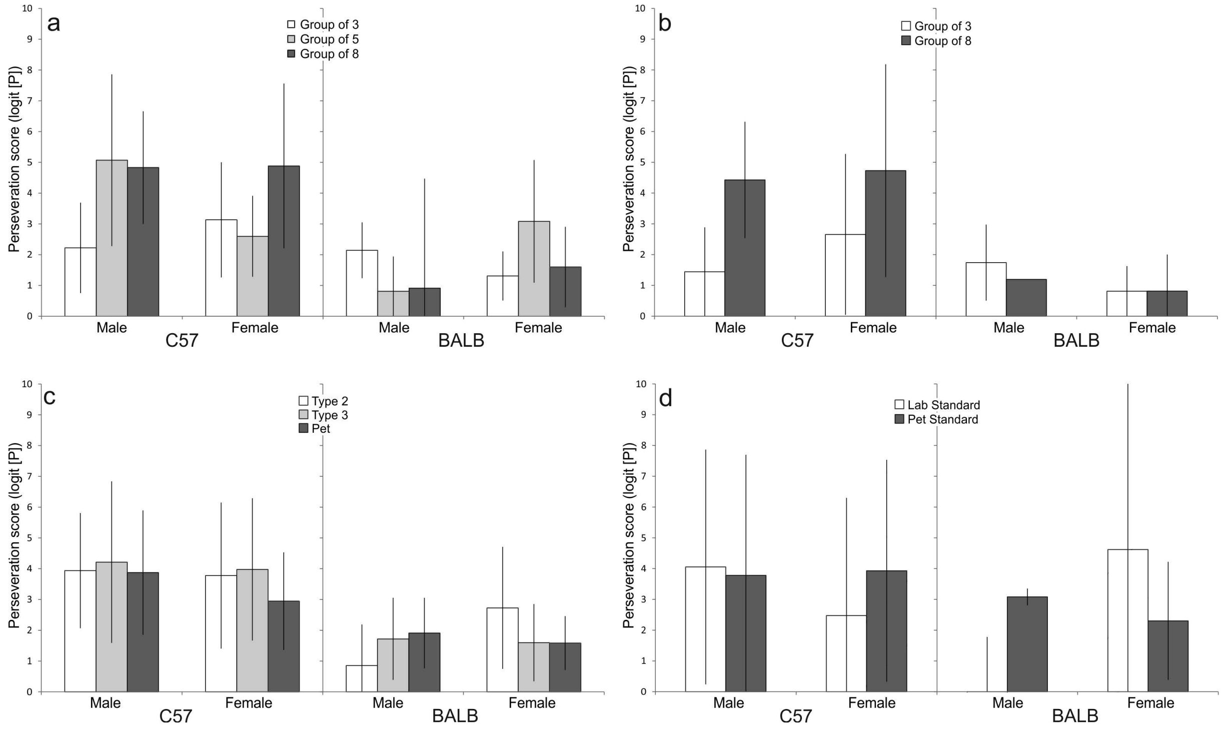

Supplementary Figure 18. Comparison of repetitions and alternations to all other sequences by: a) increased group size, controlling for floor area, b) increased group size, space allocation constant, b) increased floor area, controlling for group size and d) lab vs. pet standards.

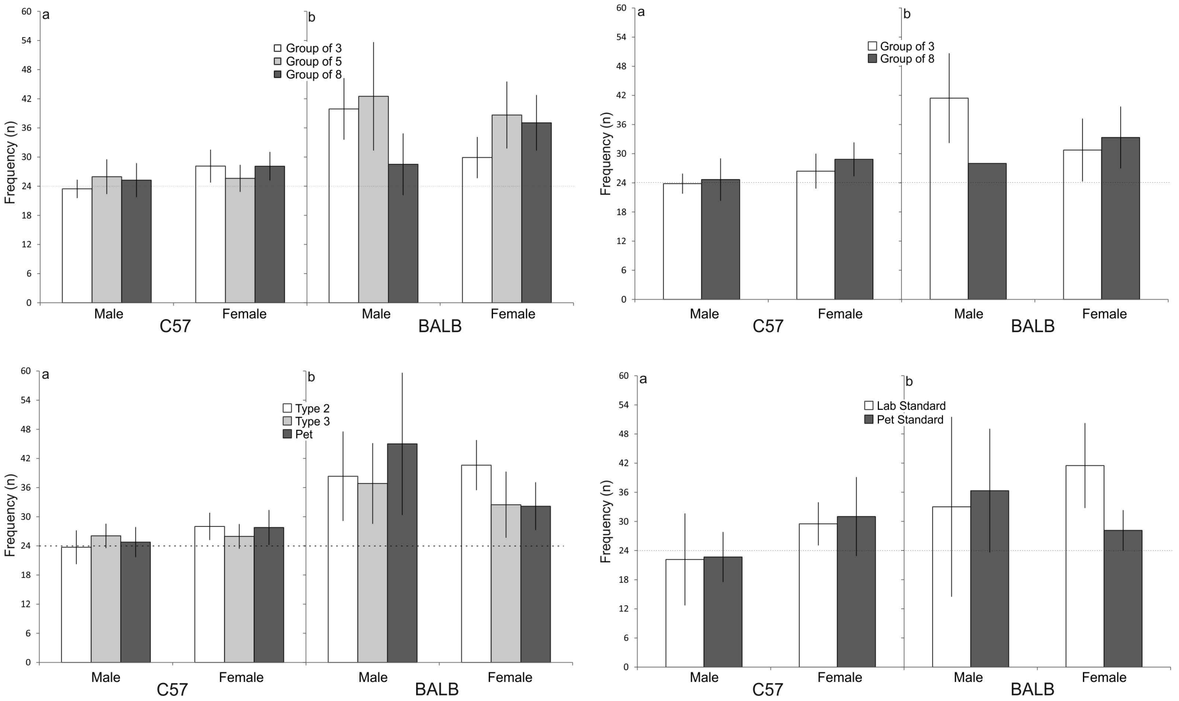

Supplementary Figure 19. Distribution of alternations and repetitions by strain

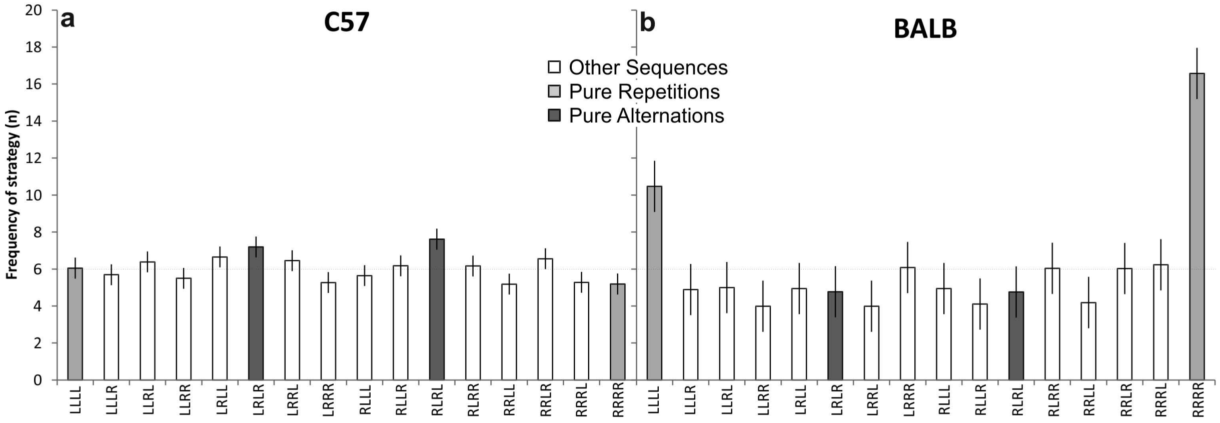

SI References

1. Hall, C. S. Emotional behavior in the rat. I. Defecation and urination as measures of individual differences in emotionality. *J. Comp. Psychol.* **18,** 385–403 (1934).

2. Walsh, R. N. & Cummins, R. A. The Open-Field Test: a critical review. *Psychol. Bull.* **83,** 482–504 (1976).

3. Belzung, C. in *Techniques in the Behavioral and Neural Sciences* **13,** 738–749 (1999).

4. Archer, J. Tests for emotionality in rats and mice: A review. *Anim. Behav.* **21,** 205–235 (1973).

5. Denenberg, V. H. Open-Field Behavior in the Rat: What does it mean? *Ann. N. Y. Acad. Sci.* **159,** 852–859 (1969).

6. Carola, V., D'Olimpio, F., Brunamonti, E., Mangia, F. & Renzi, P. Evaluation of the elevated plus-maze and open-field tests for the assessment of anxiety-related behaviour in inbred mice. *Behav. Brain Res.* **134,** 49–57 (2002).

7. Whimbey, A. E. & Denenberg, V. Two independent behavioral dimensions in open-field performance. *J. Comp. Physiol. Psychol.* **63,** 500–504 (1967).

8. Martin-Arenas, F. J. & Pintado, C. O. Results of the Open Field Test at different light intensities in C57 mice. in *Measuring Behavior* 0–4 (2014).

9. Bailoo, J. D., Bohlen, M. O. & Wahlsten, D. L. The precision of video and photocell tracking systems and the elimination of tracking errors with infrared backlighting. *J. Neurosci. Methods* **188,** 45–52 (2010).

10. Novak, J., Bailoo, J. D., Melotti, L. & Würbel, H. Effect of Cage-Induced Stereotypies on Measures of Affective State and Recurrent Perseveration in CD-1 and C57BL/6

Mice. *PLoS One* **11,** e0153203 (2016).

11. Garner, J. P. & Mason, G. J. Evidence for a relationship between cage stereotypies and behavioural disinhibition in laboratory rodents. *Behav. Brain Res.* **136,** 83–92 (2002).

12. Garner, J. P., Mason, G. J. & Smith, R. Stereotypic route-tracing in experimentally caged songbirds correlates with general behavioural disinhibition. *Anim. Behav.* **66,** 711–727 (2003).

13. Frith, C. D. *The Cognitive Neuropsychology of Schizophrenia (Classic Edition).* (Psychology Press, 2015).

14. Ridley, R. M. The psychology of perseverative and stereotyped behaviour. *Prog. Neurobiol.* **44,** 221–231 (1994).

15. Garner, J. P., Meehan, C. L. & Mench, J. A. Stereotypies in caged parrots, schizophrenia and autism: evidence for a common mechanism. *Behav. Brain Res.* **145,** 125–34 (2003).

16. Gross, A. N., Engel, A. K. J., Richter, S. H., Garner, J. P. & Würbel, H. Cage-induced stereotypies in female ICR CD-1 mice do not correlate with recurrent perseveration. *Behav. Brain Res.* **216,** 613–620 (2011).
